# Rapid, biochemical tagging of cellular activity history in vivo

**DOI:** 10.1038/s41592-024-02375-7

**Published:** 2024-08-05

**Authors:** Run Zhang, Maribel Anguiano, Isak K. Aarrestad, Sophia Lin, Joshua Chandra, Sruti S. Vadde, David E. Olson, Christina K. Kim

**Affiliations:** 1 Biomedical Engineering Graduate Group, University of California, Davis, Davis, CA USA; 2 Center for Neuroscience, University of California, Davis, Davis, CA USA; 3 Neuroscience Graduate Group, University of California, Davis, Davis, CA USA; 4 Institute for Psychedelics and Neurotherapeutics, University of California, Davis, Davis, CA USA; 5 Department of Neurology, University of California, Davis, Sacramento, CA USA; 6 Department of Chemistry, University of California, Davis, Davis, CA USA; 7 Department of Biochemistry and Molecular Medicine, University of California, Davis, Sacramento, CA USA

**Keywords:** Molecular engineering, Neuroscience, Mouse

## Abstract

Intracellular calcium (Ca^2+^) is ubiquitous to cell signaling across biology. While existing fluorescent sensors and reporters can detect activated cells with elevated Ca^2+^ levels, these approaches require implants to deliver light to deep tissue, precluding their noninvasive use in freely behaving animals. Here we engineered an enzyme-catalyzed approach that rapidly and biochemically tags cells with elevated Ca^2+^ in vivo. Ca^2+^-activated split-TurboID (CaST) labels activated cells within 10 min with an exogenously delivered biotin molecule. The enzymatic signal increases with Ca^2+^ concentration and biotin labeling time, demonstrating that CaST is a time-gated integrator of total Ca^2+^ activity. Furthermore, the CaST readout can be performed immediately after activity labeling, in contrast to transcriptional reporters that require hours to produce signal. These capabilities allowed us to apply CaST to tag prefrontal cortex neurons activated by psilocybin, and to correlate the CaST signal with psilocybin-induced head-twitch responses in untethered mice.

## Abstract (web summary)

CaST is a Ca^2+^-activated version of split-TurboID. The tool allows labeling active neurons quickly, simply by administration of exogenous biotin, thus enabling the study of behaviors that would be impaired by hardware required for the use of other, light-dependent tools.

## Main

Dynamic changes in intracellular ion concentrations allow cells to respond and adapt to their local environment, ultimately contributing to the normal physiological functioning of organisms. For example, neurons, the basic functional units of the brain, can be activated by various external stimuli or pharmacological compounds, leading to rapid fluctuations in intracellular Ca^2+^ concentrations. Thus, activity among complex neural networks can be measured using cellular changes in Ca^2+^ levels as a direct proxy for neuronal firing. Genetically encodable Ca^2+^ indicators have transformed our ability to record neural activity in awake and behaving animals^1–4^. However, a major limitation of fluorescent sensors is that their readout is transient, and they generally require invasive methods to gain optical access to deep brain structures. This can make it challenging to couple the activity history of a given neuron with its numerous other cellular properties (for example, precise spatial localization, RNA expression or protein expression).

To overcome this issue, previous efforts have focused on designing orthogonal transcriptional reporters (FLARE^5^, FLiCRE^6^, Cal-Light^7^) or fluorescent proteins (CaMPARI^8^) that can stably mark activated cells undergoing high intracellular Ca^2+^ levels. However, these approaches implement light-sensitive proteins that require blue^9^ or ultraviolet^10^ light to restrict the time window of activity labeling in cells. This requirement hampers their scalability in deep brain regions, or in body areas where fibers for light delivery cannot be implanted. Alternative stable tagging approaches include immediate early gene (IEG)-based transcriptional reporters (TRAP2 and tetTag^11–13^), which utilize a drug injection instead of light to gate the activity labeling window. However, while IEG activity has been linked to neural activity in many cell types^14^, it is not nearly as universal a readout as Ca^2+^ is. Furthermore, the slow onset of IEG expression limits the ability to immediately tag and identify neurons activated during a specific time window. This is compounded by the fact that all transcription-based activity reporters take several hours (~6–18 h^6,15^) before sufficient levels of the reporter protein can be detected. Thus, there is a need for a strategy that enables noninvasive and rapid activity-dependent labeling of cells.

We designed an activity-dependent enzyme that can attach a small, biochemical handle to activated cells exhibiting high intracellular Ca^2+^. Our strategy was to reengineer and repurpose a proximity-labeling enzyme, split-TurboID^16^, to report increased intracellular Ca^2+^ in living cells by tagging proteins with an exogenously delivered biotin molecule. Proximity-labeling enzymes such as split-TurboID^16^ (and its predecessors, BioID^17^ and TurboID^18^) have been traditionally used to biotinylate nearby, statically present proteins for downstream enrichment and analysis over long periods (typically 4–7 days in vivo). But they have not been engineered to detect dynamic changes in intracellular ion concentrations. Our design, Ca^2+^-activated split-TurboID (CaST), enzymatically tags activated neurons within brief, user-defined time windows of exogenous biotin delivery. The biotinylated proteins can then immediately be read out using any existing method for biotin detection. Because the biotin molecule is permeable to both the cell and the blood–brain barrier^19,20^, this facilitates its application in living organisms.

## Results

### Design and optimization of CaST

**Fig. 1:**
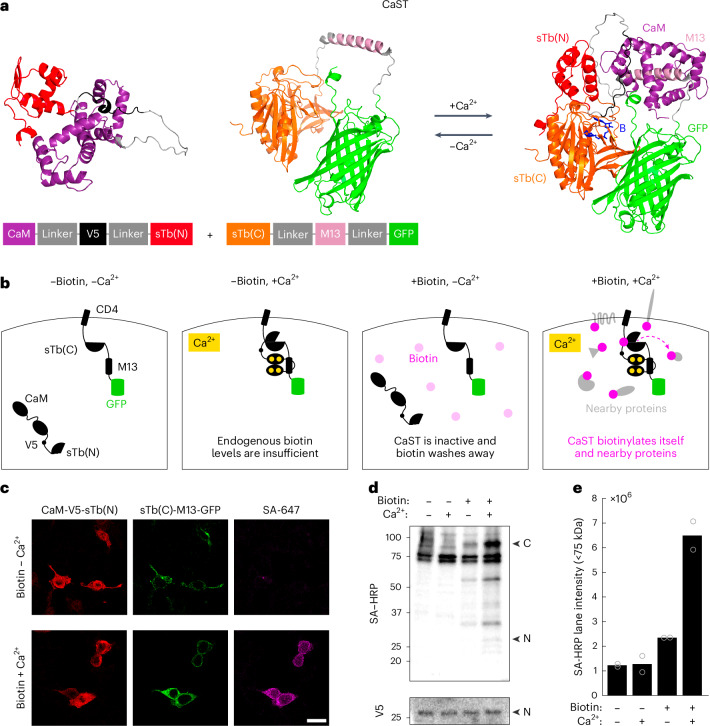
Design of CaST. **a**, AlphaFold2 (refs. ^56,57^) prediction of the protein structures for the two halves of CaST either in isolation (left; as expected in the absence of Ca^2+^) or in complex (right; as expected in the presence of high Ca^2+^). The two CaST components reversibly reconstitute in a Ca^2+^-dependent manner. The predicted biotin binding site is shown in blue. **b**, Schematic of CaST design for expression in HEK cells. The component with sTb(C)-M13-GFP is tethered to the membrane via the transmembrane domain of a CD4 cell membrane protein^58^, while the CaM-V5 epitope tag^59^-sTb(N) component is expressed throughout the cytosol. CaST only tags proteins when cells are treated with biotin and exhibit elevated intracellular Ca^2+^. **c**, Example confocal images of HEK cells transfected with both components of CaST and treated with biotin ± Ca^2+^ for 30 min. Cells were washed, fixed and stained with anti-V5 and SA-647. The anti-V5 signal stains the CaM-sTb(N) component (left), while the GFP fluorescence shows the CD4-sTb(C)-M13 component (middle). Biotinylation of proteins is detected by SA-647 staining (right). Scale bar, 20 µm. **d**, HEK cells were transfected with CaST and treated with ±50 µM biotin and ±Ca^2+^ (5 mM CaCl_2_ and 1 µM ionomycin) for 30 min. Cells were then washed with Dulbecco’s phosphate buffered saline (DPBS), and whole-cell lysates were collected and analyzed using a western blot stained with streptavidin–horseradish peroxidase (SA–HRP) or anti-V5/HRP. ‘N’ indicates the expected size of the CaM-V5-sTb(N) fragment, while ‘C’ indicates the expected size of the CD4-sTb(C)-M13-GFP fragment. **e**, Quantification of biotinylated proteins present in western blot lanes of the experiment shown in **d**. Two independent biological replicates were quantified. The entire lane below the 75-kDa endogenously biotinylated bands was included in the quantification (sum of the total raw intensity pixel values). A line plot profile spanning the entire blot is shown in Extended Data Fig. 2. Source data

The basic CaST design tethers the Ca^2+^-binding protein calmodulin (CaM) and a CaM-binding synthetic peptide M13 variant to either inactive half of split-TurboID (Fig. 1a). We postulated that under high cytosolic Ca^2+^ concentrations, the CaM fragment would be recruited to M13, resulting in the reconstitution and activation of split-TurboID. Upon simultaneous biotin supplementation, the reconstituted split-TurboID would then biotinylate itself and nearby proteins^16^, in a Ca^2+^-dependent manner. High Ca^2+^ alone should not result in signal, as the endogenous biotin levels are too low to result in substantial protein biotinylation; and exogenous biotin alone should not result in signal, as the split-TurboID fragments remain separated and inactive (Fig. 1b). Thus, CaST can act as a coincidence detector of both exogenous biotin and high intracellular Ca^2+^.

**Extended Data Fig. 1:**
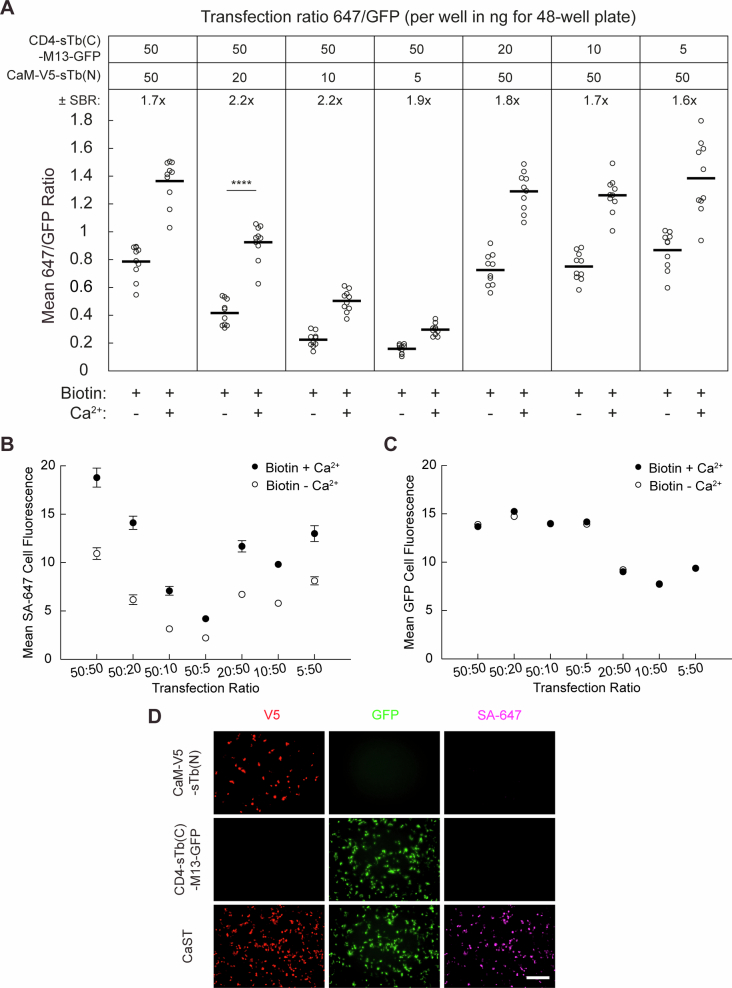
Comparison of different CaST transfection ratios of fragments. **A)** HEK cells were transfected with different ratios of the CaST fragments indicated above in ng for 48-well plate and incubated overnight. Cells were treated with 50 µM biotin and ± Ca^2+^ (5 mM CaCl_2_ and 1 µM ionomycin) for 30 minutes. The ± Ca^2+^ SBR and the FOV averages of the SA647/GFP fluorescence ratio per cell are shown. The ratio of 50:20 (CD4-sTb(C)-M13-GFP:CaM-V5-sTb(N)) had a significant 2.2-fold ± Ca^2+^ SBR (*n* = 10 FOVs per condition; *P* = 1.1**e-5**, U = 0, two-tailed Mann-Whitney U test). **B,C)** The FOV average of the SA-647 cell fluorescence (**B**) and the GFP cell fluorescence (**C**) was calculated for each transfection ratio (CD4-sTb(C)-M13-GFP:CaM-V5-sTb(N)) after biotin ± Ca^2+^ stimulation (*n* = 10 FOVs per condition). Data are plotted as mean ± s.e.m. **D)** HEK cells were transfected with either the Cam-V5-sTb(N) fragment (top), the CD4-sTb(C)-M13-GFP fragment (middle), or both fragments of CaST together (bottom). Cells were treated with 50 µM biotin and Ca^2+^ (5 mM CaCl_2_ and 1 µM ionomycin) for 30 minutes, then washed, stained, and imaged. Data were replicated across 12 FOVs. Scale bar, 300 µm. ****P < 0.0001. Source data

Initially, we tested various approaches to tether CaM and M13 to either of the split-TurboID fragments, sTb(N) and sTb(C). We transfected four different versions of the tool into human embryonic kidney (HEK) 293T cells, with different conformational arrangements and subcellular localizations of the proteins (Supplementary Fig. 1a). We first treated the cells with a combination of biotin with or without Ca^2+^ and an ionophore for 30 min, and then we fixed and stained the cells for biotinylated proteins using streptavidin conjugated to Alexa Fluor 647 (SA-647). We quantified both the green fluorescent protein (GFP) and SA-647 fluorescence for each cell and calculated their ratio (SA-647/GFP) to normalize for differences in expression levels across cells or experimental conditions. Importantly, all four versions had no SA-647 signal in the absence of exogenously delivered biotin. Of the four versions, we found that a membrane-tethered CD4-sTb(C)-M13-GFP with a cytosolic CaM-V5-sTb(N) resulted in the highest signal-to-background ratio (SBR) of biotin ± Ca^2+^ tagging (Supplementary Fig. 1b). We subsequently designated this optimized construct as our final CaST design (Fig. 1b). We also showed that a 5:2 transfection ratio of the two CaST fragments (CD4-sTb(C)-M13-GFP to CaM-V5-sTb(N)) yielded the highest SBR of all ratios tested (Extended Data Fig. 1a–c). We performed subsequent characterizations using this optimal transfection ratio.

**Extended Data Fig. 2:**
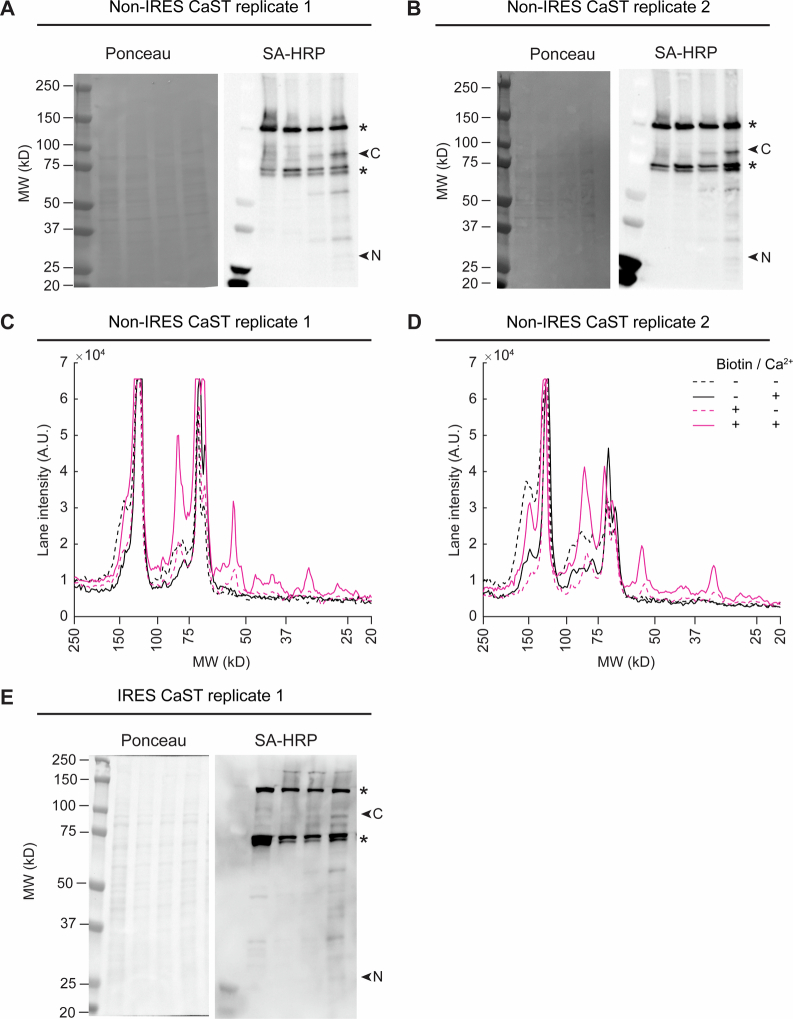
Additional western blot analysis of CaST labeling. **A)** Ponceau staining and raw image of the full SA-HRP blot from Fig. 1d. **B)** Biological replicate of experiment in panel **A**. **C,D)** Line plot profile of the entire lane of the raw blot shown in panels **A** and **B** (drawn vertically from the 250 kB to the 20 kB ladder line and centered horizontally within each lane). **E)** Additional biological replicate experiment of panels **A**,**B**, except using CaST-IRES. Cells were otherwise treated identically. *indicate bands showing endogenously biotinylated proteins. “N” indicates the expected size of the CaM-V5-sTb(N) fragment, while “C” indicates the expected size of the CD4-sTb(C)-M13-GFP fragment. Source data

Immunohistochemistry and confocal imaging showed the expression of both fragments of the tool in HEK cells, confirming that the low SA-647 signal in negative control conditions is not due to a lack of fragment coexpression (Fig. 1c). As expected, purposefully omitting either fragment of CaST in the presence of biotin and Ca^2+^ resulted in no biotinylation signal (Extended Data Fig. 1d). Western blot analysis confirmed that CaST-transfected HEK239T cells treated with biotin and Ca^2+^ drove biotinylation across an array of nearby proteins, compared to negative control conditions (Fig. 1d,e and Extended Data Fig. 2a–d).

### Characterization of CaST labeling in HEK cells

**Fig. 2:**
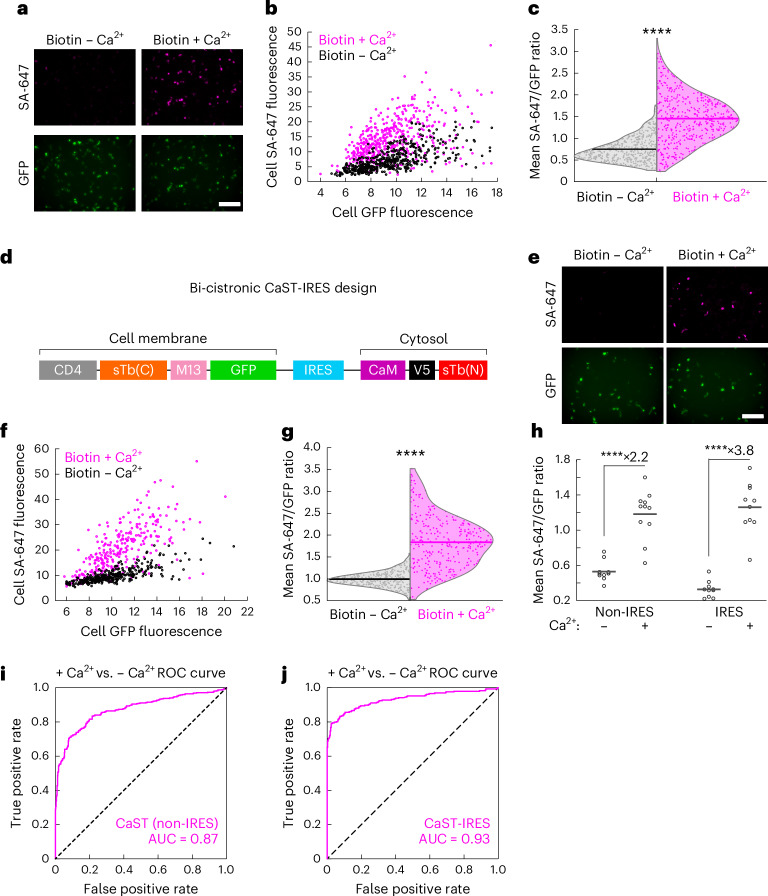
Quantification of CaST’s performance. **a**, Example images of HEK cells transfected with CaST and treated with 50 µM biotin ± Ca^2+^ (5 mM CaCl_2_ and 1 µM ionomycin) for 30 min. Top row shows SA-647 staining of biotinylated proteins. Bottom row shows CD4-sTb(C)-M13-GFP. **b**, Scatterplot of the mean SA-647 versus mean GFP fluorescence calculated for every GFP^+^ cell detected across 11 FOVs treated with either biotin + Ca^2+^ (*n* = 451 cells; two-tailed Pearson’s *R* = 0.50, *P* = 6.2 × 10^−30^) or biotin − Ca^2+^ (*n* = 473 cells; two-tailed Pearson’s *R* = 0.69, *P* = 2.6 × 10^−67^). **c**, Violin plots showing the distributions of the mean SA-647/GFP fluorescence ratio per cell from data in **b** (*P* = 2.0 × 10^−85^, *U* = 27,234, two-tailed Mann–Whitney *U* test). **d**, Schematic of the bi-cistronic CaST-IRES construct design. **e**, Example images of HEK cells transfected with CaST-IRES and treated with biotin ± Ca^2+^ for 30 min, as in **a**. **f**, Scatterplot of the mean SA-647 versus mean GFP fluorescence calculated for each GFP^+^ cell detected across 10 FOVs treated with either biotin + Ca^2+^ (*n* = 293 cells; two-tailed Pearson’s *R* = 0.72, *P* = 1.5 × 10^−47^) or biotin − Ca^2+^ (*n* = 332 cells; two-tailed Pearson’s *R* = 0.78, *P* = 3.7 × 10^−69^). **g**, Violin plots showing the distributions of the mean SA-647/GFP fluorescence ratio per cell from data in **f** (*P* = 3.1 × 10^−^^77^, *U* = 6,732, two-tailed Mann–Whitney *U* test). **h**, The FOV averages of the SA-647/GFP fluorescence ratio per cell from the non-IRES data shown in **b** (*n* = 11 FOVs per condition; *P* = 1.1 × 10^−^^5^, *U* = 2, two-tailed Mann–Whitney *U* test), and the IRES data shown in **f** (*n* = 10 FOVs per condition; *P* = 1.1 × 10^−^^5^, *U* = 0, two-tailed Mann–Whitney *U* test). **i**,**j**, ROC curves for distinguishing Ca^2+^-treated versus non-treated cell populations based on CaST non-IRES cells from **c** (**i**; AUC = 0.87, *P* = 2.0 × 10^−^^85^, Wilson/Brown method) and CaST-IRES-transfected cells from **g** (**j**; AUC = 0.93, *P* = 3.1 × 10^−^^77^, Wilson/Brown method). All scale bars, 300 µm. ****P < 0.0001. Source data

To further quantify the extent of biotin-dependent and Ca^2+^-dependent CaST labeling, we performed fluorescence imaging across multiple fields of view (FOVs) in CaST-transfected HEK cells treated with biotin ± Ca^2+^ (Fig. 2a). Single-cell analysis confirmed that the Ca^2+^-dependent increase in SA-647 labeling occurred across cells with varying GFP expression levels, showing that it is not due to differences in expression levels of the tool across conditions (Fig. 2b). The distributions of normalized SA-647/GFP fluorescence also differed between cells treated with or without Ca^2+^ (Fig. 2c). These results demonstrate that CaST robustly detects elevated intracellular Ca^2+^ levels in living cells.

To optimize CaST delivery, we concatenated its two fragments into a bi-cistronic vector containing either a porcine teschovirus 2A peptide (P2A) coding sequence or an internal ribosome entry site (IRES). P2A and IRES are well-established strategies for coexpressing multiple proteins from a single promoter^21,22^, ensuring that each cell expresses both fragments of CaST. We found that both CaST-P2A and CaST-IRES exhibited higher SA-647/GFP labeling with biotin and Ca^2+^ compared to with biotin alone; but the IRES version resulted in a higher biotin ± Ca^2+^ SBR (5-fold, compared to 2.7-fold; Supplementary Fig. 2). Studies have reported that the IRES motif lowers the expression level of the second component relative to the first^23^; this may explain why this strategy performs better than P2A, given our data showing an optimal transfection ratio of 5:2 for the two separate components of CaST (Extended Data Fig. 1a). For these reasons, we chose to further characterize the CaST-IRES version (Fig. 2d–g).

**Extended Data Fig. 3:**
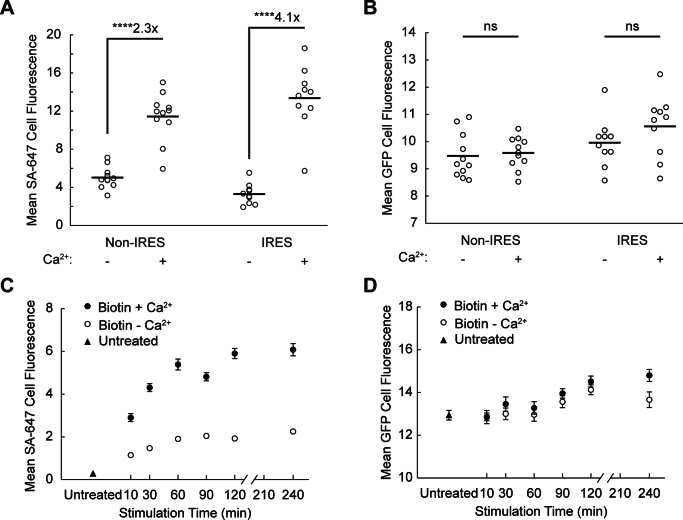
Additional characterization of CaST in HEK cells. **A)** The FOV averages of the SA-647 fluorescence per cell from the non-IRES data shown in Fig. 2b, and the IRES data shown in Fig. 2f. The non-IRES version exhibited a Ca^2+^-dependent SBR of 2.3x (*n* = 11 FOVs per condition; *P* = 1.1e-5, U = 2, two-tailed Mann-Whitney U test). The Ca^2+^-dependent SBR for the IRES version was 4.1x (*n* = 10 FOVs per condition; *P* = 1.1e-5, U = 0, two-tailed Mann-Whitney U test). **B)** The FOV averages of the GFP cell fluorescence per cell from the non-IRES data shown in Fig. 2b (*n* = 11 FOVs per condition; *P* = 0.562, U = 51, two-tailed Mann-Whitney U test), and the IRES data shown in Fig. 2f (*n* = 10 FOVs per condition; *P* = 0.165, U = 31, two-tailed Mann-Whitney U test). **C,D)** The FOV average of the SA-647 (**C**) or GFP (**D**) cell fluorescence was calculated for each stimulation duration for data shown in Fig. 3e,f (*n* = 10 FOVs per condition). Cells were transfected with CaST-IRES. The untreated condition is shown on the left. Data are plotted as mean ± s.e.m. ****P < 0.0001, ns, not significant. Source data

Western blot analysis confirmed that CaST-IRES cells exhibited elevated biotinylation signal compared to negative control conditions (Extended Data Fig. 2e). In addition, immunofluorescence analysis showed that in comparison to the non-IRES version, CaST-IRES resulted in a larger calcium-dependent fold-change in both the mean SA-647/GFP cell ratio and the mean SA-647 cell fluorescence (likely due to more controlled protein expression levels of the two components; Fig. 2h and Extended Data Fig. 3a,b). We also performed receiver operating characteristic (ROC) analyses of the SA-647/GFP ratios to evaluate CaST’s ability to discriminate between individual Ca^2+^-treated and non-treated cells. We determined an area under the curve (AUC) of 0.87 for non-IRES CaST, and an AUC of 0.93 for CaST-IRES, indicating that both versions can robustly distinguish activated versus non-activated cells (Fig. 2i,j).

### Temporal resolution, Ca^2+^ sensitivity and time integration of CaST

**Fig. 3:**
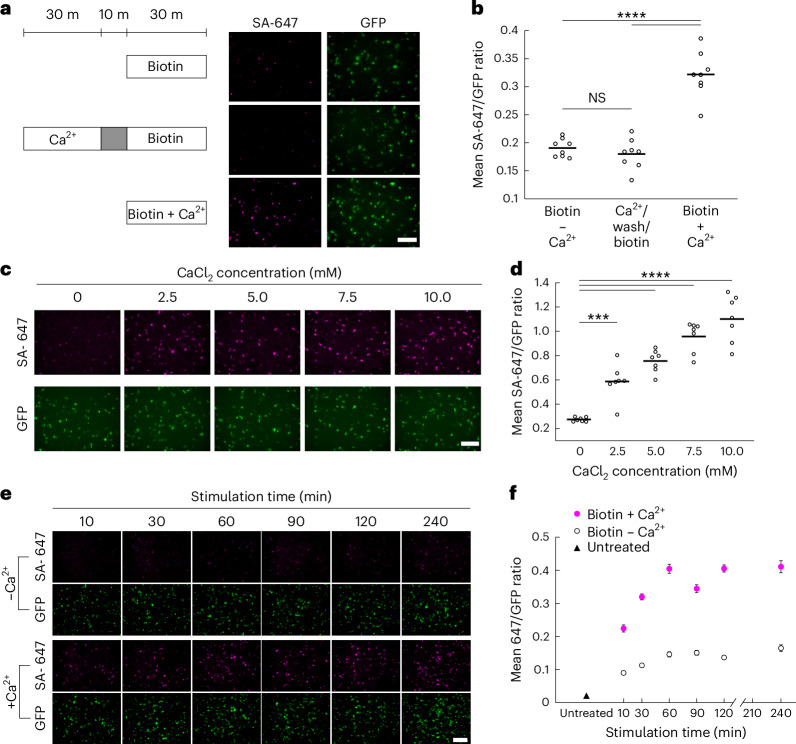
Characterization of reversibility, Ca^2+^ sensitivity and time integration of CaST. **a**, To test the split enzyme’s reversibility, HEK cells were transfected with CaST-IRES and treated with biotin alone for 30 min (top), with Ca^2+^ for 30 min followed by a 10-min wash and then biotin for 30 min (middle), or with biotin + Ca^2+^ simultaneously for 30 min (bottom). Example images are shown for all three conditions with SA-647 staining of biotin and GFP expression of CaST-IRES. Scale bar, 300 µm. **b**, The FOV averages of the SA-647/GFP fluorescence ratio per cell for the three conditions shown in **a** (*n* = 8 FOVs per condition; biotin − Ca^2+^ versus Ca^2+^/wash/biotin: *P* = 0.75; biotin − Ca^2+^ versus biotin + Ca^2+^: *P* = 4.9 × 10^−8^; Ca^2+^/wash/biotin versus biotin + Ca^2+^: *P* = 1.3 × 10^−8^; Tukey’s post hoc multiple-comparison’s test following a one-way analysis of variance (ANOVA), *F*_2,21_ = 56.37, *P* = 3.6 × 10^−9^). **c**, Example FOVs of HEK cells transfected with CaST-IRES and treated with biotin and increasing concentrations of CaCl_2_ (and 1 µM ionomycin). **d**, The FOV averages of the SA-647/GFP fluorescence ratio per cell for the CaCl_2_ concentrations shown in **c** (*n* = 7 FOVs per condition; 0 mM versus 2.5 mM: *P* = 7.6 × 10^−4^; 0 mM versus 5 mM: *P* = 8.8 × 10^−7^; 0 mM versus 7.5 mM: *P* = 5.5 × 10^−10^; 0 mM versus 10 mM: *P* = 5.4 × 10^−12^; Tukey’s post hoc multiple-comparison’s test following a one-way ANOVA, *F*_4,30_ = 44.07, *P* = 3.8 × 10^−12^). The FOV average SA-647/GFP ratios were linearly correlated with CaCl_2_ concentration (two-tailed Pearson’s correlation coefficient *R* = 0.99, *P* = 0.001). **e**, Example FOVs of HEK cells transfected with CaST-IRES and treated with 50 µM biotin ± Ca^2+^ (5 mM CaCl_2_ and 1 µM ionomycin) for different durations. **f**, The mean FOV averages of the SA-647/GFP fluorescence ratio per cell for the different stimulation times shown in **e** (*n* = 10 FOVs per condition). The untreated condition is shown on the left. Data are plotted as the mean ± s.e.m. All scale bars, 300 µm. ***P < 0.001, ****P < 0.0001. NS, not significant. Source data

One important requisite of our design is that both the Ca^2+^-sensing and split-TurboID reconstitution are reversible. This is so that enzymes activated during high Ca^2+^ before the desired biotin labeling window can split back into inactive fragments once the cytosolic Ca^2+^ returns to resting levels. To test for the reversibility of CaST, we treated HEK cells expressing CaST-IRES with Ca^2+^ for 30 min, washed the cells over the course of 10 min and then delivered biotin for 30 min. We directly compared this condition to CaST-IRES cells treated with biotin alone, or with biotin and Ca^2+^. Cells treated with biotin after removal of Ca^2+^ exhibited no biotinylation, similar to the negative control (Fig. 3a,b). This demonstrates that CaST has a temporal resolution for detecting intracellular Ca^2+^ on the order of 10 min (meaning it can ignore Ca^2+^ events that occur 10 min before the desired biotin labeling window).

Next, we asked whether the CaST signal is correlated to the levels of intracellular Ca^2+^ present in cells. We conducted a Ca^2+^ titration experiment in which we treated cells with increasing concentrations of calcium chloride (CaCl_2_) in the medium (along with 1 µM ionomycin and biotin) for 30 min. Our results demonstrated a monotonically increasing SA-647/GFP signal from 2.5 mM to 10 mM CaCl_2_, with a linear correlation to Ca^2+^ concentration over this range (Fig. 3c,d).

We also conducted temporal integration experiments to evaluate the labeling of CaST over different time exposures to a fixed Ca^2+^ concentration. We treated CaST-IRES cells with biotin ± Ca^2+^ for increasing durations of time. We found that a 10-min stimulation period is sufficient to induce elevated SA-647/GFP labeling over background (Fig. 3e,f). The amount of labeling increased with longer stimulation times, saturating around 1 h (Extended Data Fig. 3c,d). These results show that CaST acts as an integrator of Ca^2+^, both in terms of detecting increasing Ca^2+^ concentration, and increasing duration of Ca^2+^ exposure.

### Direct comparison of CaST to existing technologies

**Fig. 4:**
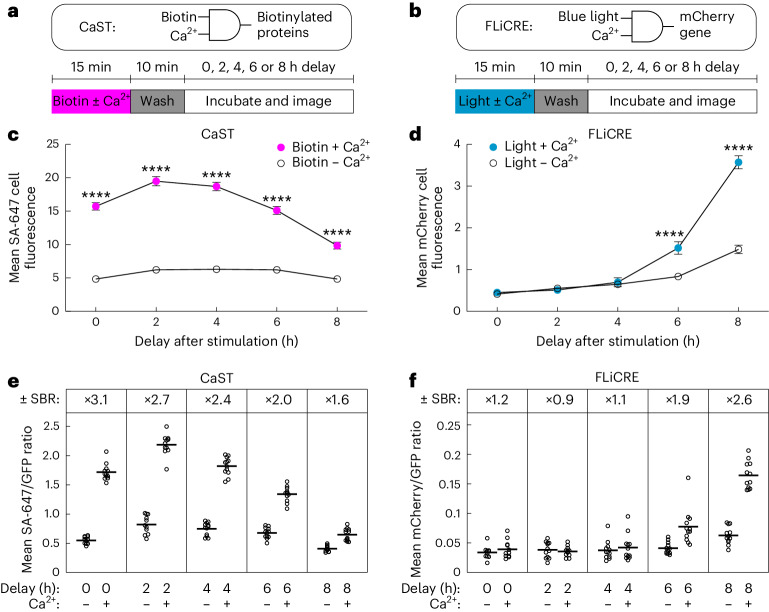
Comparison of CaST to an existing technology, FLiCRE. **a**,**b**, Schematics of CaST (**a**) and FLiCRE (**b**) as AND logic gates, and the experimental paradigms used to test the time course of labeling detection after biotin + Ca^2+^ (CaST) and light + Ca^2+^ (FLiCRE) stimulation. Cells were transfected with either CaST-IRES or FLiCRE (Extended Data Fig. 4a) components. **c**, For CaST, the FOV average of the SA-647 cell fluorescence was calculated following a variable delay period after biotin ± Ca^2+^ stimulation (*n* = 12 FOVs for conditions with 0, 4, 6 and 8 h delay; *n* = 11 FOVs for conditions with 2 h delay; 0 h: *P* = 1.0 × 10^−30^; 2 h: *P* = 3.0 × 10^−36^; 4 h: *P* = 2.4 × 10^−35^; 6 h: *P* = 3.0 × 10^−24^; 8 h: *P* = 1.4 × 10^−10^; Sidak’s post hoc multiple-comparison’s test following a two-way ANOVA, *F*_4,108_ = 25.94, *P* = 4.5 × 10^−15^). **d**, For FLiCRE, the FOV average of the UAS::mCherry cell fluorescence was calculated following a variable delay period after light ± Ca^2+^ stimulation (*n* = 11 FOVs for conditions with 0 h delay; *n* = 12 FOVs for conditions with 2, 4, 6 and 8 h delay; 6 h: *P* = 4.6 × 10^−5^; 8 h: *P* = 2.4 × 10^−28^; Sidak’s post hoc multiple-comparison’s test following a two-way ANOVA, *F*_4,108_ = 46.46, *P* = 1.2 × 10^−22^). **e**,**f**, The ±Ca^2+^ SBR of normalized reporter expression is shown for both CaST (**e**) and FLiCRE (**f**). For CaST, the SA-647 fluorescence was divided by the GFP fluorescence (*n* = 12 FOVs for conditions with 0, 4, 6 and 8 h delay; *n* = 11 FOVs for conditions with 2 h delay). For FLiCRE, the UAS::mCherry fluorescence was divided by the GFP fluorescence (*n* = 11 FOVs for conditions with 0 h delay; *n* = 12 FOVs for conditions with 2, 4, 6 and 8 h delay). Data are plotted as the mean ± s.e.m. in **c** and **d**. ****P < 0.0001. Source data

**Extended Data Fig. 4:**
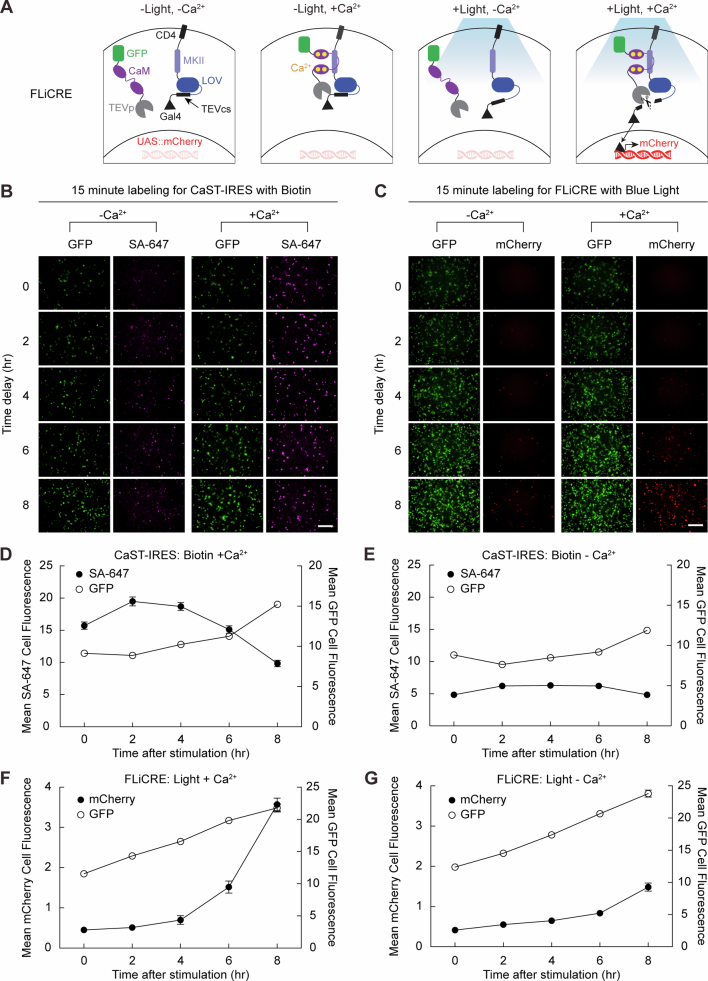
Comparison of CaST to FLiCRE. **A)** Schematic of FLiCRE as a light- and Ca^2+^-dependent transcriptional reporter. A TEV protease (TEVp) is tethered to GFP-CaM and expressed in the cytosol. A CD4-MKII-LOV-TEVcs-Gal4 fusion is expressed at the membrane. In the dark, the LOV protein cages the TEV cleavage site (TEVcs), protecting it from the TEVp. When there is high intracellular Ca^2+^, CaM-M13 interact to bring the TEVp nearby the TEVcs. However, only when blue light is simultaneously delivered, will the TEVcs become uncaged and available for cleavage. With both blue light and high intracellular Ca^2+^, the TEVp will cut the TEVcs, and the released Gal4 then enters the nucleus to drive expression of the UAS reporter gene. **B,C)** Example FOVs for CaST (**B**) and FLiCRE (**C**) experiments quantified in Fig. 4c–f. Scale bar, 300 µm. **D,E)** For CaST, the FOV average of the SA-647 cell fluorescence and the GFP cell fluorescence was calculated following a variable delay period after biotin + Ca^2+^ (**D**) or biotin - Ca^2+^ (**E**) stimulation (*n* = 12 FOVs for conditions with 0, 4, 6, 8 hr delay time after stimulation; *n* = 11 FOVs for conditions with 2 hr delay time after stimulation). **F,G)** For FLiCRE, the FOV average of the UAS-mCherry cell fluorescence and the GFP cell fluorescence was calculated following a variable delay period after light + Ca^2+^ (**D**) or light - Ca^2+^ (**E**) stimulation (*n* = 11 FOVs for conditions with 0 hr delay time after stimulation; *n* = 12 FOVs for conditions with 2, 4, 6, 8 hr delay time after stimulation). Data are plotted as mean ± s.e.m. in **D**–**G**. Source data

Compared to existing Ca^2+^-dependent integrators, CaST has two major advantages: it is noninvasive (using biotin instead of light), and it acts on rapid timescales (tags cells within minutes rather than hours). We directly compared the performance of CaST against an existing light-dependent and Ca^2+^-dependent integrator, FLiCRE^6^. FLiCRE works via a protease-mediated release of a non-native transcription factor (for example, Gal4) in the presence of blue light and Ca^2+^. This released transcription factor then enters the nucleus to drive expression of a modular reporter gene, such as a fluorescent protein (Extended Data Fig. 4a). While it has fast labeling kinetics (it can detect 30 s of blue light and elevated Ca^2+^), its readout kinetics are slow, requiring transcription and translation of the reporter gene that can take hours to accumulate. Here we transfected HEK cells overnight with either CaST-IRES or FLiCRE and treated cells for 15 min with either biotin ± Ca^2+^ or light ± Ca^2+^, respectively. We then fixed cells immediately or 2, 4, 6 or 8 h after the stimulation, and imaged the reporter expression in each case (SA-647 for CaST, and UAS::mCherry for FLiCRE; Fig. 4a,b and Extended Data Fig. 4b,c). Across all time points measured, we observed an increase in CaST SA-647 labeling in conditions treated with biotin and Ca^2+^ compared to with biotin alone; however, an increase in FLiCRE UAS::mCherry reporter expression was only apparent starting 6 h after the light and Ca^2+^ stimulation (Fig. 4c,d). We calculated the biotin ± Ca^2+^ and light ± Ca^2+^ SBRs by normalizing the SA-647 or UAS::mCherry expression against an expression marker for each respective tool (GFP; Fig. 4e,f and Extended Data Fig. 4d–g). Note that for the cells treated with biotin and Ca^2+^, the decrease in the SA-647/GFP ratio over the 8 h is due to both a decrease in SA-647 signal (protein turnover) and an increase in CaST GFP expression (Extended Data Fig. 4d,e).

To ensure the use of optimal labeling parameters for evaluating FLiCRE, we also stimulated cells at higher expression levels of each tool (48 h after transfection). We observed an increase in nonspecific background labeling for both CaST-IRES and FLiCRE, even immediately after stimulation (Supplementary Fig. 3a,b). Nonetheless, we were still able to observe a difference between biotin ± Ca^2+^ groups immediately after stimulation with CaST-IRES (Supplementary Fig. 3c–e). In contrast, FLiCRE showed no differences between light ± Ca^2+^ groups, even at 8 h and 24 h after stimulation, due to nonspecific background activation caused by high expression levels (Supplementary Fig. 3f–h). Previous studies have also reported that the performance of FLiCRE^6^ and related protease-dependent tools^24^ can suffer at high expression levels. These findings demonstrate both the immediacy of labeling, and robustness at various expression levels, of CaST compared to protease-driven transcriptional tools such as FLiCRE.

### Application of CaST in cultured neurons

**Fig. 5:**
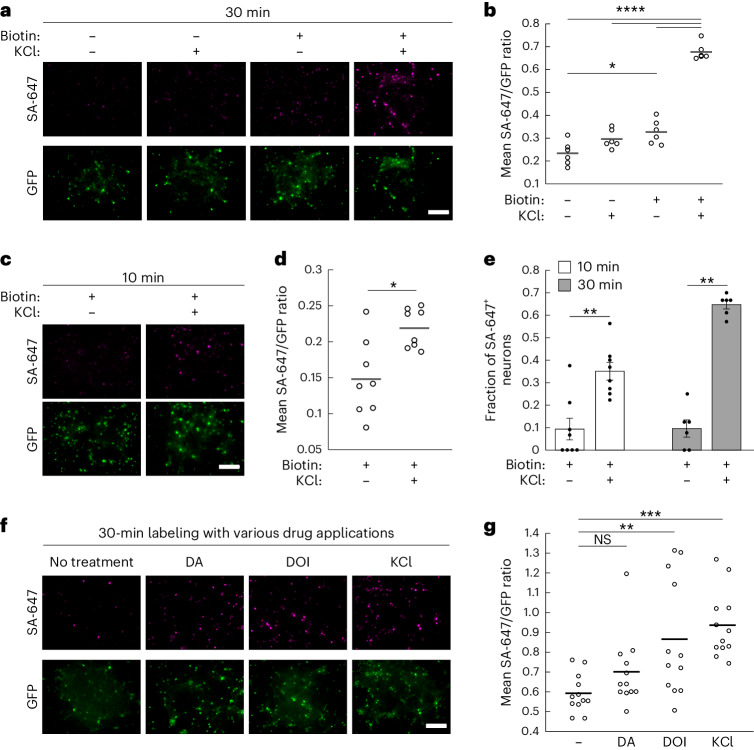
CaST performance in cultured neurons. **a**, Example FOVs of cultured rat hippocampal neurons infected with AAV2/1-Synapsin-CD4-sTb(C)-M13-GFP and AAV2/1-Synapsin-CaM-sTb(N) viruses and stimulated with ±biotin and ±KCl for 30 min. **b**, The FOV averages of the SA-647/GFP fluorescence ratio per cell for the data shown in **a** (*n* = 6 FOVs per condition; −biotin −KCl versus +biotin +KCl: *P* = 1.8 × 10^−12^; −biotin +KCl versus +biotin +KCl: *P* = 3.0 × 10^−11^; +biotin −KCl versus +biotin +KCl: *P* = 1.4 × 10^−10^; −biotin −KCl versus +biotin −KCl: *P* = 0.013; Sidak’s post hoc multiple-comparison’s test following a two-way ANOVA, *F*_1,20_ = 59.43, *P* = 2.1 × 10^−7^). **c**, Example FOVs of rat hippocampal neurons infected with CaST as in **a** but stimulated with biotin ± KCl for only 10 min. **d**, The FOV averages of the SA-647/GFP fluorescence ratio per cell for the data shown in **c** (*n* = 8 FOVs per condition; *P* = 0.015, *U* = 9, two-tailed Mann–Whitney *U* test). **e**, Fraction of all GFP^+^ neurons that are also SA-647^+^ (defined as having an SA-647 fluorescence value greater than the 90th percentile of neurons in the biotin − KCl group). Data are quantified for the 10-min labeling experiment shown in **c** (*n* = 8 FOVs per condition; *P* = 0.003, *U* = 5, two-tailed Mann–Whitney *U* test) and for a replicated 30-min labeling experiment shown in Extended Data Fig. 5b,c (*n* = 6 FOVs per condition; *P* = 0.002, *U* = 0, two-tailed Mann–Whitney *U* test). Data are plotted as the mean ± s.e.m. **f**, Example FOVs of rat hippocampal neurons infected with CaST as in **a** and treated with 50 µM biotin and 10 µM DA, 10 µM DOI or 30 mM KCl for 30 min. **g**, The FOV averages of the SA-647/GFP fluorescence ratio per cell for the conditions shown in **f** (*n* = 12 FOVs per condition; −KCl versus DA: *P* = 0.547; −KCl versus DOI: *P* = 0.008; −KCl versus KCl: *P* = 6.5 × 10^−4^, Tukey’s post hoc multiple-comparison’s test following a one-way ANOVA, *F*_3,44_ = 7.373, *P* = 4.2 × 10^−4^). All scale bars, 300 µm. *P < 0.05, **P < 0.01, ***P < 0.001, ****P < 0.0001. Source data

**Extended Data Fig. 5:**
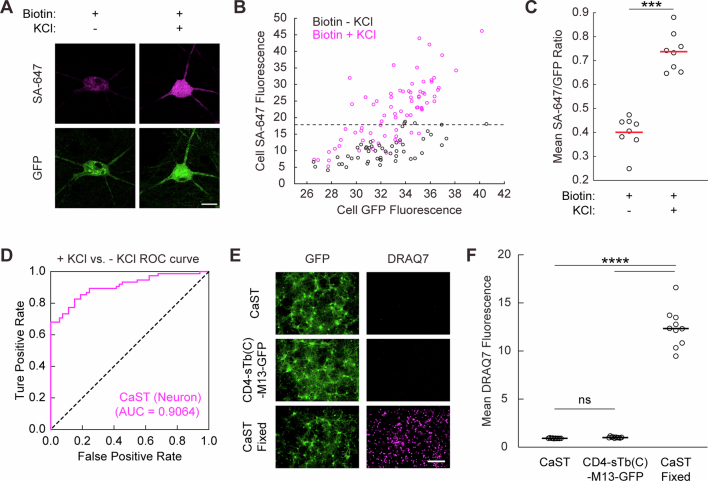
Additional characterization of CaST in neurons. **A)** Example confocal images of cultured rat hippocampal neurons infected with both components of CaST. Neurons were treated with 50 µM biotin ± 30 mM KCl for 30 minutes. They were then washed, fixed, and stained for SA-647. Scale bar, 20 µm. **B)** Scatter plot of the SA-647 versus GFP fluorescence calculated for each GFP+ neuron detected across FOVs treated with biotin - KCl (*n* = 53 neurons pooled from 8 FOVs) or biotin + KCl (*n* = 75 neurons pooled from 8 FOVs). Dashed line indicates the 90^th^ percentile of SA-647 fluorescence values of all neurons in the biotin - KCl group. **C)** The FOV averages of the SA-647/GFP fluorescence ratio per cell from the data shown in panel **B** (*n* = 8 FOVs per condition; *P* = 1.6e-4, U = 0, two-tailed Mann-Whitney U test). **D)** ROC curve for distinguishing KCl-treated vs. non-treated neuron populations based on the SA-647/GFP ratios from panel **B** (AUC = 0.91, *P* = 5.5e-15, Wilson/Brown’s method). **E)** Example FOV images of CaST expressing neuron viability experiment. Neurons expressing both fragments of CaST together (top) or the CD4-sTb(C)-M13-GFP fragment (middle) were stained with DRAQ7 at a final concentration of 3 µM at DIV 19 before fixation. Neurons expressing both fragments of CaST (bottom) were fixed, permeabilized, and stained with DRAQ7 at DIV 19. Scale bar, 300 µm. **F)** The FOV averages of the DRAQ7 fluorescence for the 3 conditions shown in panel **D** (*n* = 10 FOVs per condition; CaST versus CD4-sTb(C)-M13-GFP: *P* = 0.9831; CaST versus CaST Fixed: *P* = 8.0e-15; CD4-sTb(C)-M13-GFP versus CaST Fixed: *P* = 8.0e-15, Tukey’s post-hoc multiple comparison’s test following a 1-way ANOVA, F_2,27_ = 326.3, *P* = 1.2e-19). ***P < 0.001, ****P < 0.0001, ns, not significant. Source data

We then asked whether CaST could detect elevated intracellular Ca^2+^ in cultured rat hippocampal neurons. We expressed the two-component version of CaST using adeno-associated viruses (AAVs) to obtain the maximal expression levels, and stimulated neurons using potassium chloride (KCl) in the presence of biotin for 30 min. Immunofluorescence imaging confirmed that CaST robustly tagged activated neurons treated with biotin and KCl, compared to negative control conditions (Fig. 5a,b and Extended Data Fig. 5a). Stimulation for only 10 min was also sufficient to drive elevated CaST labeling (Fig. 5c,d). Quantitative analysis of individual GFP^+^ neurons expressing CaST showed that ~35% of GFP^+^ neurons exhibited strong SA-647 labeling in the 10-min biotin and KCl condition (thresholded as described in Extended Data Fig. 5b), compared to ~10% labeled in the biotin-alone condition (Fig. 5e). In neurons stimulated for 30 min, ~65% of GFP^+^ neurons exhibited strong SA-647 labeling, compared to ~10% labeled in the biotin-alone condition (Fig. 5e and Extended Data Fig. 5b,c). ROC analysis showed that under the 30-min condition, CaST could distinguish KCl-stimulated versus unstimulated neurons with an AUC of 0.91 (Extended Data Fig. 5d). DRAQ7 staining showed no apparent cytotoxicity before stimulation in neurons transduced with CaST (Extended Data Fig. 5e,f).

**Extended Data Fig. 6:**
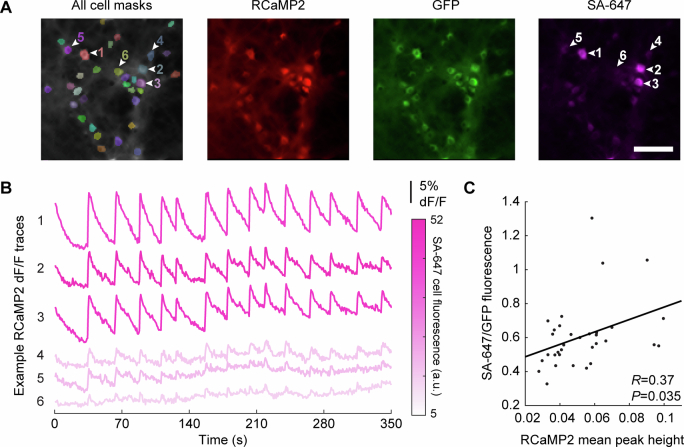
Simultaneous RCaMP2 imaging and CaST labeling in neurons. **A)** Example FOV images of neurons co-infected with AAV2/1-Synapsin-RCaMP2 and AAV2/1-Synapsin-CaST viruses, following mild stimulation (50% media change) and 50 µM biotin treatment for 30 minutes. Post-hoc RCaMP2 and CaST labeling is shown for all identified cell masks during RCaMP2 imaging (shown as colored overlays to the left). Numbered arrows indicate locations from which traces were extracted for panel **B**. Scale bar, 100 µm. **B)** RCaMP2 dF/F fluorescence traces of example neurons from panel **A**, during a ~5-minute recording following treatment. Traces are colored according to their SA-647 cell fluorescence intensity value. **C)** Scatter plot showing a linear correlation between SA-647/GFP fluorescence ratio calculated for each GFP+ neuron detected, and the mean peak height during the RCaMP2 recording for each cell (*n* = 33 cells; two-tailed Pearson’s correlation coefficient *R* = 0.37, *P* = 0.035). Source data

**Extended Data Fig. 7:**
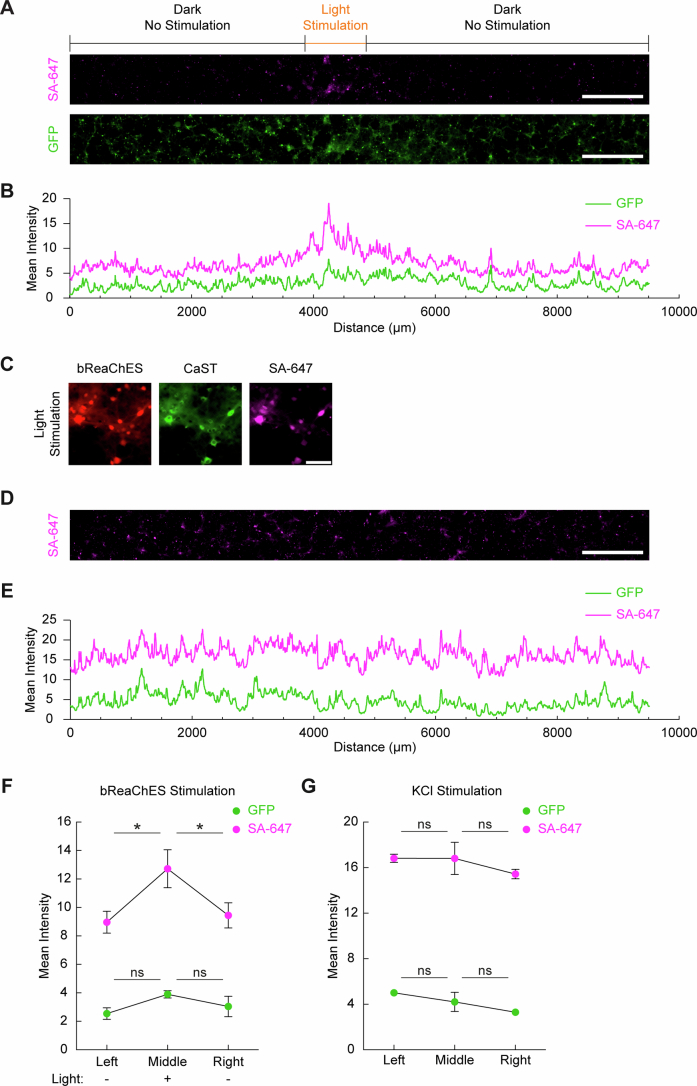
CaST specificity using targeted optogenetic stimulation. **A)** Example fluorescence images for neurons co-infected with an excitatory opsin, AAV2/1-Synapsin-mCherry-P2A-bReaChES, and AAV2/1-Synapsin-CaST. 50 µM biotin and orange light was delivered for 30 minutes through a ~1 mm wide slit to the bottom of the culture dish. 50 µM APV and 20 µM NBQX were added at the time of light stimulation to reduce synchronized neuron firing. Scale bar, 1000 µm. **B)** Quantification of the mean SA-647 and GFP fluorescence intensity, averaged vertically across the entire FOV images shown in panel **A**. **C)** Example zoom-in images of the light-stimulated region in panel **A** showing neurons co-expressing bReaChES and CaST. Scale bar, 100 µm. **D)** Example SA-647 image for CaST-expressing neurons with whole dish KCl treatment as a non-spatial control. Scale bar, 1000 µm. **E)** Quantification of the mean SA-647 and GFP fluorescence intensity, averaged vertically across the entire FOV shown in panel **D**. **F)** Mean SA-647 and GFP fluorescence intensity, averaged vertically and binned across 1 mm horizontal sections to the left, middle, or right of the light stimulation gap (For SA-647, *n* = 3 FOVs per condition; Left versus Middle: *P* = 0.0281; Middle versus Right: *P* = 0.0449, Šídák’s post-hoc multiple comparison’s test following a 2-way ANOVA, F_2,4_ = 12.94, *P* = 0.0179) (For GFP, *n* = 3 FOVs per condition; Left versus Middle: *P* = 0.1645; Middle versus Right: *P* = 0.4352, Šídák’s post-hoc multiple comparison’s test following a 2-way ANOVA, F_2,4_ = 3.536, *P* = 0.1305). **G)** Same analysis as in panel **F**, except for a whole dish KCl stimulation non-spatial control (For SA-647, *n* = 3 FOVs per condition; Left versus Middle: *P* = 1.0; Middle versus Right: *P* = 0.6731, Šídák’s post-hoc multiple comparison’s test following a 2-way ANOVA, F_2,4_ = 0.9006, *P* = 0.4754) (For GFP, *n* = 3 FOVs per condition; Left versus Middle: *P* = 0.7328; Middle versus Right: *P* = 0.6682, Šídák’s post-hoc multiple comparison’s test following a 2-way ANOVA, F_2,4_ = 2.446, *P* = 0.2023). Data are plotted as mean ± s.e.m. in **F** and **G**. *P < 0.05, ns, not significant. Source data

Next, we explored the relationship between intracellular Ca^2+^ changes and CaST labeling using the real-time fluorescent calcium indicator RCaMP2 (ref. ^4^). We co-infected RCaMP2 and CaST AAVs in neurons, and mildly stimulated them using a media change. We found that neurons exhibiting greater Ca^2+^ changes in response to stimulation (quantified by the RCaMP2 mean d*F/F* peak height) also displayed stronger SA-647/GFP CaST labeling (Extended Data Fig. 6). This demonstrates on a cell-by-cell basis that CaST labeling is correlated to intracellular Ca^2+^ level changes. To additionally demonstrate the specificity of CaST labeling, we used a red-shifted optogenetic cation channel, bReaChES^25^, to achieve spatially targeted neuron stimulation using orange light. We selectively stimulated neurons within a narrow ~1-mm width slit through the bottom of the culture dish. We observed an increased SA-647/GFP cell ratio only within the subregion of the FOV exposed to orange light, while KCl stimulation resulted in uniform CaST biotinylation across the dish (Extended Data Fig. 7).

**Extended Data Fig. 8:**
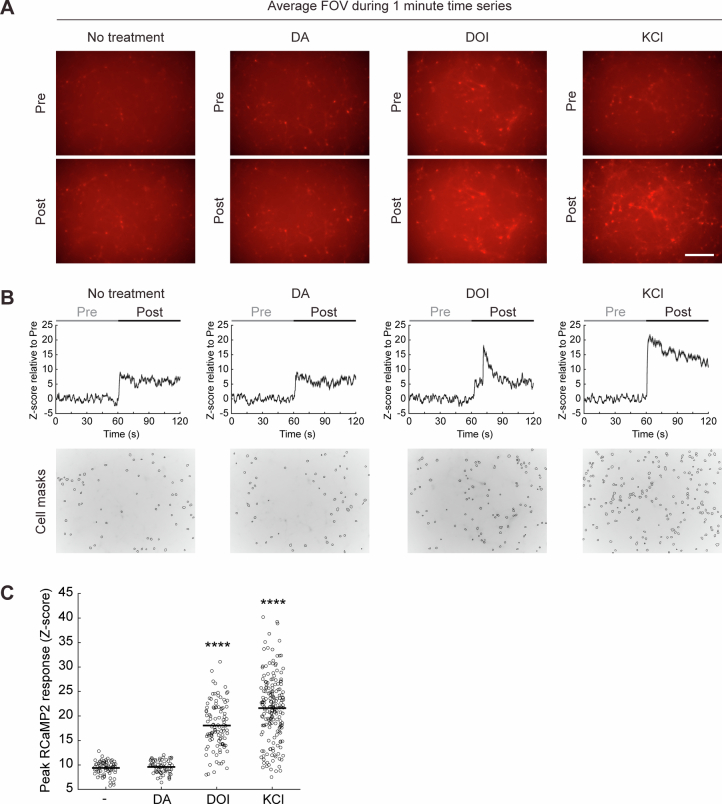
RCaMP2 calcium imaging during various drug applications. **A)** Average FOV images of neurons infected with AAV2/1-Synapsin-RCamp2, taken from the entire RCaMP2 baseline recording (“Pre”) or post-treatment recording (“Post”). Each recording was 1 minute long. Neurons were treated with 50 µM biotin and vehicle, 10 µM dopamine (DA), 10 µM DOI, or 30 mM KCl for 30 minutes. Scale bar, 300 µm. **B)** Top: average pre- and post-treatment fluorescence traces for all identified neurons in the FOV (Z-scored relative to the baseline “pre” period for each cell). Bottom: cell masks identified for each FOV shown in panel **A**. Data are plotted as mean ± s.e.m. with the shaded regions indicating the s.e.m. **C)** Peak neuron responses during the post-treatment recordings. Only DOI and KCl treatment drove a larger peak RCaMP2 response compared to the vehicle control (*n* = 69 cells for vehicle, 62 cells for DA, 104 cells for DOI, and 189 cells for KCl treatment; No KCl versus DOI: *P* = 2.3e-13; No KCl versus KCl: *P* = 2.3e-13, Tukey’s post-hoc multiple comparison’s test following a 1-way ANOVA, F_3,420_ = 152.4, *P* = 7.8e-67). ****P < 0.0001. Source data

Lastly, we investigated the potential for CaST to detect elevated intracellular Ca^2+^ in response to physiological stimuli, such as exposure to neuromodulators or pharmacological agents. We treated neurons with biotin and either 10 µM dopamine (DA) or 10 µM 2,5-dimethoxy-4-iodoamphetamine (DOI; a serotonin 5-HT type 2A/C receptor agonist), for 30 min. Both DA receptors and 5-HT_2A_ receptors are known to be expressed in rat hippocampal neurons^26–28^. DA receptors are either G_i_- or G_s_-protein-coupled receptors, while 5-HT_2A_ receptors are G_q_-protein-coupled receptors^29,30^. Whereas activation of G_i_/G_s_-protein signaling primarily modulates cAMP with variable effects on intracellular Ca^2+^ levels, G_q_-protein signaling should directly elevate intracellular Ca^2+^ levels^31,32^. We found that DA treatment did not increase SA-647 labeling relative to a biotin-alone vehicle control; however, biotin and DOI drove an increase in SA-647 labeling (Fig. 5f,g). RCaMP2 imaging in neurons treated under experimentally matched conditions confirmed that both DOI and KCl (but not DA) induce an increase in Ca^2+^ activity (Extended Data Fig. 8). These results demonstrate that CaST can be applied to reveal differential responses to pharmacological compounds among a population of neurons containing multiple neuronal subtypes and expressing different receptors.

### Identifying psilocybin-activated neurons during head twitch

We next applied CaST in vivo to record cellular activity in behavioral contexts that have previously been challenging to record from with existing methods. Psychedelics are an emerging class of therapeutics that are known to promote neuroplasticity in the prefrontal cortex^33–35^ and produce positive behavioral adaptations in animal models of neuropsychiatric disorders^36–39^. These compounds also drive hallucinations in humans; therefore, a major area of research is to understand whether the hallucinogenic and therapeutic aspects of psychoplastogens can be decoupled^40^. The main hallucinogenic behavioral correlate of psychedelic drugs in animal models is the head-twitch response (HTR)^41,42^—a rhythmic rotational head movement that is tightly coupled to 5-HT_2A_ receptor activation and correlated with hallucinogenic potential in humans^42^. Critically, the HTR measurement requires free movement of the animal’s head, precluding its measurement in head-fixed rodents under a microscope during cellular-resolution neuronal recordings. We posited that CaST could be applied to directly correlate cellular neuronal activity with the psychedelic-induced HTR in vivo.

We used CaST to measure how psilocybin, a potent and therapeutically relevant psychedelic, modulates population activity in the medial prefrontal cortex (mPFC) of untethered mice during the simultaneous measurement of the HTR. Notably, there have been conflicting reports as to whether 5-HT_2A_ receptor agonists increase^43–45^ or decrease^46,47^ population neuronal activity in the cortex. This could be in part due to the different sensitivities in the previous recording methods used.

**Fig. 6:**
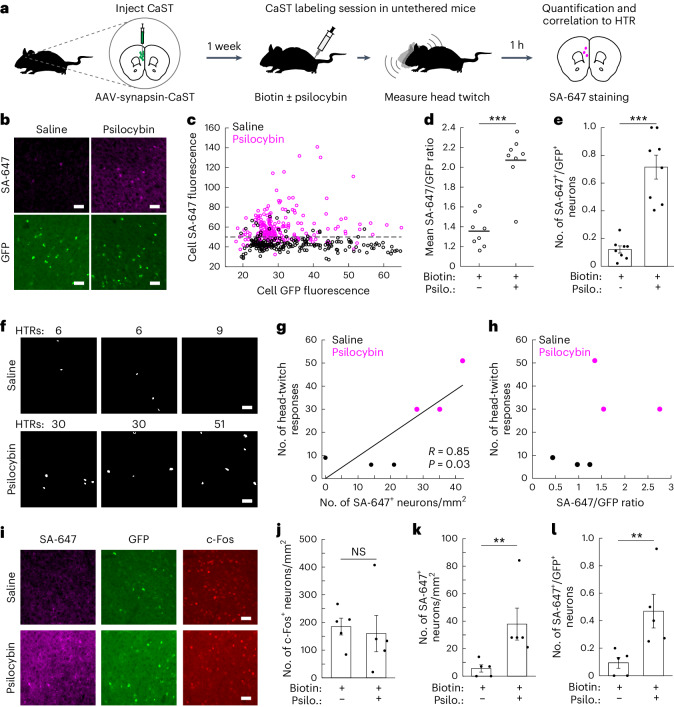
Noninvasive identification of psilocybin-activated neurons in vivo. **a**, Schematic for using CaST to tag psilocybin-activated neurons during HTR measurement. **b**, Example mPFC images of SA-647 and CaST GFP fluorescence, for mice injected with biotin + saline, or biotin + psilocybin. **c**, Mean SA-647 versus GFP fluorescence for each GFP^+^ neuron detected in biotin + saline-injected mice (*n* = 218 neurons from 3 mice) or biotin + psilocybin-injected mice (*n* = 220 neurons from 3 mice). The horizontal dashed line indicates the 90th percentile threshold value of all SA-647 neurons in the biotin + saline group. **d**, FOV averages of the SA-647/GFP fluorescence ratios from **c** (*n* = 8 FOVs pooled from 3 mice in both conditions; *P* = 6.2 × 10^−4^, *U* = 38, two-tailed Mann–Whitney *U* test). **e**, Fraction of all GFP^+^ neurons that are SA-647^+^ (thresholded using the dashed line in **c**; *n* = 8 FOVs pooled from 3 mice in both conditions; *P* = 1.6 × 10^−4^, *U* = 36, two-tailed Mann–Whitney *U* test). **f**, Cell masks of SA-647^+^ mPFC neurons identified during HTR measurements. FOVs with the same number of HTRs were taken from the same mice, but from independent CaST injections on opposite hemispheres. **g**, Number of HTRs versus the number of SA-647^+^ neurons per mm^2^ for data shown in **f** (*n* = 6 FOVs from independent CaST injections; two-tailed Pearson’s correlation coefficient *R* = 0.85, *P* = 0.03). **h**, Number of HTRs versus the mean cell SA-647/GFP ratio for data shown in **f**. **i**, Example mPFC images of CaST GFP, SA-647 staining and c-Fos staining in mice treated with biotin + saline, or biotin + psilocybin. **j**–**l**, Number of c-Fos^+^ neurons per mm^2^ (**j**), SA-647^+^ neurons per mm^2^ (**k**) or SA-647^+^ divided by GFP^+^ neurons per mm^2^ per FOV (**l**), in mice injected with biotin + saline versus biotin + psilocybin (*n* = 5 mice per condition; *P* = 0.42, *U* = 8, two-tailed Mann–Whitney *U* test (**j**); *P* = 0.0079, *U* = 0, two-tailed Mann–Whitney *U* test (**k**); *P* = 0.0079, *U* = 0, two-tailed Mann–Whitney *U* test (**l**)). All scale bars, 50 µm. Data are plotted as the mean ± s.e.m. in **e** and **j**–**l**. **P < 0.01, ***P < 0.001. Psilo., psilocybin. Source data

**Extended Data Fig. 9:**
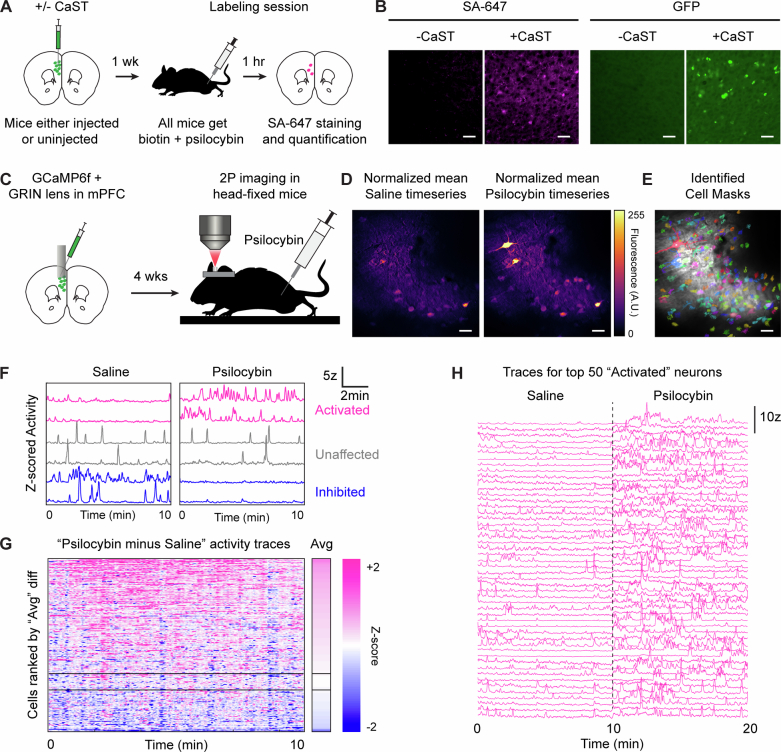
Controls and validation for in vivo CaST labeling. **A)** Schematic of control and experimental conditions. Both wildtype mice not expressing CaST, and wildtype mice injected with CaST in mPFC, were treated with 24 mg/kg biotin + 3 mg/kg psilocybin for 1 hour, and then sacrificed for histology. **B)** Example FOVs of wildtype mice not expressing CaST (-CaST) or expressing CaST (+CaST) that were injected with biotin and psilocybin. The -CaST control was replicated across two uninjected mice. **C)** Schematic for using real-time imaging to identify psilocybin-activated neurons in the mPFC. AAV5-CaMKIIa-GCaMP6f was injected into mPFC, and a 1 mm diameter GRIN lens was implanted. 4 weeks later, mice were imaged head-fixed under a 2 P microscope during an IP injection of 5 ml/kg saline or 3 mg/kg psilocybin. **D)** Background-subtracted mean images of the FOV during a 10-minute saline recording session (left), and a 10-minute psilocybin recording session (right). Active neurons are displayed as warmer pixel colors. Experiment was replicated in two mice. **E)** Mean 2 P FOV image of the combined saline and psilocybin recordings with all identified neuron masks shown as colored overlays. **F)** Example Z-scored fluorescence traces of neurons activated by psilocybin (magenta), unaffected by psilocybin (gray), or inhibited by psilocybin (blue), compared to the saline recording. Each recording was 10 minutes long. **G)** “Psilocybin minus Saline” activity traces were calculated by subtracting the baseline saline Z-scored trace from the psilocybin Z-scored trace for each neuron. The resulting 10-minute-long trace representing the difference is plotted for each neuron in the heatmap to the left, and the average difference for each neuron is plotted as the heatmap to the right labeled “Avg” (*N* = 254 cells from 2 mice). Neurons are plotted ranked by the highest to lowest average Z-score difference. The two horizontal bars represent the thresholds for defining “Activated” versus “Inhibited” neurons (>+0.05 = “Activated”, <−0.05 = “Inhibited”). **H)** The activity traces for the top 50 ranked “Activated” neurons from panel **G** are shown during the saline and psilocybin recordings (separated by a dashed vertical line). All scale bars, 50 µm. Source data

We expressed CaST viruses under a pan-neuronal synapsin promoter in the mPFC to identify what percentage of neurons are activated during acute psilocybin injection. Mice expressing CaST received a single intraperitoneal (i.p.) injection of biotin and saline, or biotin and psilocybin. We also recorded video of the mice to quantify the number of head twitches displayed following the drug treatment. One hour later, we euthanized the mice, and stained mPFC slices with SA-647 (Fig. 6a). We first asked how psilocybin modulated mPFC neuronal activity using CaST as the readout. Immunohistochemistry showed an increase in SA-647 labeling in mice treated with psilocybin, compared to control mice (Fig. 6b). Image quantification showed that individual CaST-expressing GFP^+^ neurons in psilocybin-treated mice exhibited increased SA-647 fluorescence compared to neurons in saline-treated mice (Fig. 6c). The normalized SA-647/GFP cell ratio averaged across FOVs was also higher in psilocybin-treated versus saline-treated mice (Fig. 6d). CaST identified that ~70% of GFP^+^ neurons in the mPFC exhibited strong SA-647 labeling following psilocybin treatment (Fig. 6e). Mice not expressing CaST, but injected with biotin and psilocybin, did not exhibit SA-647 labeling (Extended Data Fig. 9a,b).

No prior reports have measured Ca^2+^ activity in the mPFC following psilocybin. Thus, to validate our finding that psilocybin activated a large fraction of mPFC neurons using CaST, we performed two-photon (2P) scanning microscopy in head-fixed mice. We injected mice in the mPFC with an AAV encoding the Ca^2+^ indicator GCaMP6f and implanted a 1.0-mm-diameter gradient-index (GRIN) lens to optically access neurons ~2.5 mm deep in the brain (Extended Data Fig. 9c). We conducted 2P imaging in mice immediately after administering saline or psilocybin (Extended Data Fig. 9d–f). Similarly to our findings with CaST, 2P imaging identified a substantial population of neurons in the mPFC activated by psilocybin (Extended Data Fig. 9g,h).

While the 2P GRIN lens imaging validated our CaST findings, this methodology inflicts damage to the surrounding tissue, and it also requires head-fixation to record activity. It is impossible to measure the HTR during head-fixed 2P imaging, and this behavior may also be hampered using miniaturized head-mounted microscopes^48^. Indeed, studies have thus far only quantified the HTR during bulk fiber photometry recordings^49^, which lacks cellular resolution. Here we were able to simultaneously record the HTR during CaST labeling in mice injected with either saline or psilocybin. We then measured the amount of neuronal activity induced in the mPFC as a function of the HTR observed (Fig. 6f). The number of SA-647^+^ neurons showed a positive correlation with the number of HTRs measured during the recordings (Fig. 6g). The mean SA-647/GFP fluorescence ratio of all neurons was also higher in mice exhibiting psilocybin-induced HTRs (Fig. 6h).

**Extended Data Fig. 10:**
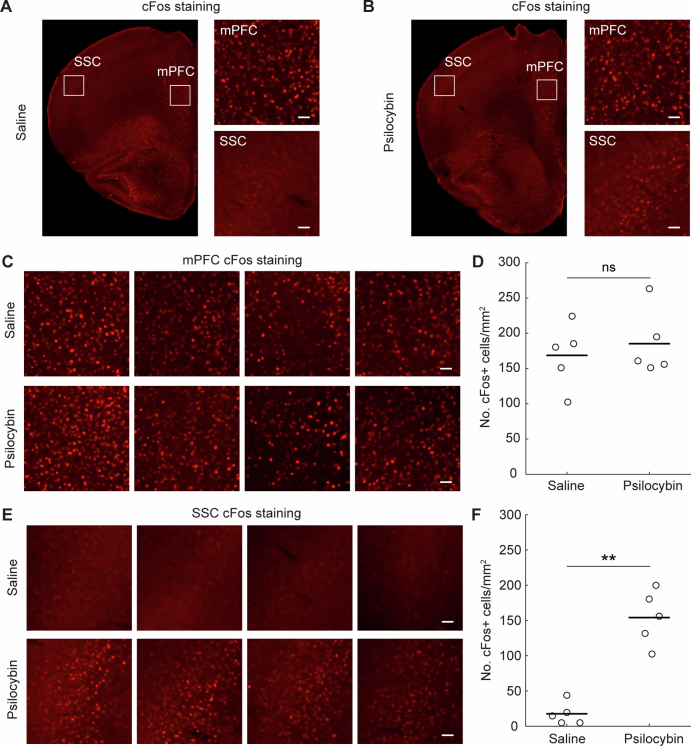
cFos-only staining in psilocybin- versus saline-injected mice. **A,B)** 2x (left) and 10x (right) images of mouse brain slices stained for cFos 1 hour after injection with saline vehicle (**A**) or 2 mg/kg psilocybin I.P. (**B**). White boxes on 2x images show location where the 10x images were taken in either the mPFC or SSC. **C)** An additional 4 example FOVs taken from different mice showing cFos staining in the mPFC after saline or psilocybin injection as described in panel **A**. **D)** Mean number of cFos+ neurons/mm^2^ in the mPFC counted from mice injected with either saline or psilocybin (*n* = 5 mice each condition; *P* = 0.73, U = 10.50, two-tailed Mann-Whitney U test). **E)** An additional 4 example FOVs taken from the same mice as in panel **C**, except showing cFos staining in the SSC after saline or psilocybin injection. **F)** Mean number of cFos+ neurons/mm^2^ in the SSC counted from mice injected with either saline or psilocybin (*n* = 5 mice each condition; *P* = 0.0079, U = 0, two-tailed Mann-Whitney U test). All scale bars, 50 µm. **P < 0.01, ns, not significant. Source data

To compare these results to the current best-in-class, activity-dependent antibody staining method compatible with untethered mice, we repeated our CaST experiment while staining for c-Fos. We found that c-Fos staining did not show a relative increase in labeling in the mPFC following psilocybin injection, due to high background staining in this region even with a saline injection (Fig. 6i,j and Extended Data Fig. 10a–d). As previously reported^45^, c-Fos staining showed increased labeling in the somatosensory cortex of psilocybin-treated mice (Extended Data Fig. 10e,f). This result confirms that there were no issues with our c-Fos staining protocol and suggests that a major limitation of c-Fos is its variability across brain regions. In contrast, CaST showed an increased number of SA-647^+^ neurons in mice treated with psilocybin compared to saline control, even when normalizing for the total number of CaST-expressing GFP^+^ cells in each FOV (Fig. 6k,l). Altogether, these results establish CaST as a sensitive technology for rapid and noninvasive tagging of activated neurons in untethered mice—providing a powerful and complementary strategy to existing molecular tools for recording and identifying activated neurons in vivo.

## Discussion

Here we demonstrate an enzymatic approach for stably tagging activated cells with a biocompatible handle both in vitro and in vivo. Our method, CaST, acts as a Ca^2+^-dependent ligase, labeling itself and nearby proteins with a biotin tag in living cells. Endogenous biotin levels in cells and in the brain are low enough that CaST requires exogenously delivered biotin to robustly tag proteins. Thus, CaST acts as a time-gated integrator of Ca^2+^ concentration, driving the accumulation of biotinylated proteins during a specified labeling period. Importantly, CaST reconstitution is reversible, and following a period of Ca^2+^ activation, it can be reset to its inactivated state within 10 min. Due to its tight temporal resolution and Ca^2+^ sensitivity, CaST could robustly detect mPFC neurons activated by psilocybin in vivo during the simultaneous measurement of the HTR (which could not be detected using c-Fos labeling).

CaST reported that psilocybin activates a large fraction of neurons in the prelimbic mPFC in mice. This was corroborated by our own secondary validation studies using single-cell Ca^2+^ imaging. A previous study in rats using in vivo electrophysiology reported that another 5-HT_2A_ receptor agonist, DOI, primarily inhibits mPFC population activity in rats^46^. It is possible that in vivo electrophysiology recordings may be biased toward more highly active neurons at baseline (missing more silent neurons that are activated by psychedelics); or that DOI and psilocybin have different effects on population activity in mPFC. Another recent study using c-Fos labeling following psilocybin injection (1 mg per kg body weight) reported that they could detect elevated c-Fos labeling in the mPFC only when using one of the two different imaging modalities that they tested^45^; in addition, our own studies here showed that we could not detect a difference in mPFC c-Fos labeling following psilocybin injection. This suggests that the neuronal activity changes driven by psilocybin in the mPFC are challenging to tag using IEG-based approaches and are better detected using Ca^2+^-based approaches such as CaST.

In contrast to existing light-gated methods for Ca^2+^-dependent labeling^5,6,8^, a major advantage of CaST is that it is essentially noninvasive, requiring only the brief i.p. injection of a biotin solution in mice, which can rapidly cross the blood–brain barrier. This is particularly advantageous in deep tissue, where light must be delivered through fiber implantation, limiting the recording area and the range of tool usage. It could also be advantageous in other areas of the body, such as the pancreas or in the spinal cord, where it is not possible to deliver blue light. As TurboID is already applicable for cell-type-specific biotin proximity labeling across the entire brain^50^, it is feasible that CaST can also be scaled as a brain-wide activity-dependent labeling tool in the future, with the use of PhP.eB^51^ viruses or transgenic lines. Although we did not observe overt cytotoxicity in neurons expressing CaST (nor has it been reported for transgenic TurboID mice^50^), additional assays of neuron physiology and cell health should be performed if expressing CaST for prolonged periods of time in the brain.

In addition, because the biotin molecule is directly attached to already expressed proteins, this enables the immediate readout of activated neurons after the labeling period with CaST. This contrasts with transcriptional-based reporters, including both Ca^2+^- and IEG-dependent systems, which require multiple hours before activated cells can be identified by the reporter RNA or protein. The immediacy of labeling, along with the scalability of CaST, would be particularly useful for registering cellular activity history with other spatial molecular imaging modalities, such as MERFISH^52^ or STARmap^53^ for in situ transcriptomics, or MALDI-IHC^54^ for spatial proteomics. Because CaST affords a stable readout of neuron activation following tissue fixation, one could apply spatial omics approaches to identify acutely induced changes in gene expression or protein localization caused specifically in this subset of activated neurons. The spatial distribution of fluorescently stained biotinylated proteins could be imaged and registered to the in situ omics data, or a streptavidin–oligonucleotide bound to biotinylated proteins could be detected during in situ sequencing^55^.

However, despite the advantages of CaST, it is important to note its limitations in comparison to existing tools. For example, some users may still require the fast labeling windows afforded by light-gated and calcium-gated tools, to tag neurons activated during acute behaviors that cannot be isolated during the relatively broad biotin labeling period. In addition, we note that the biotin labeling is an ‘end-point experiment’ where cells must be fixed and stained for imaging or collected and lysed for protein analysis. Although biotinylated cells can be subjected to subsequent downstream molecular or chemical analysis, proximity labeling itself does not enable further manipulation or genetic access to biotin-tagged neurons. Consequently, CaST is intended to complement, rather than replace, existing tools that can activate induced transcription factors and drive the expression of proteins such as opsins for downstream neuronal manipulation. Finally, there are several considerations for using CaST in vivo, including the relative time window of tagging based on the biotin injection, the strength of the behavioral stimulus required to induce strong enough CaST labeling and the need for negative control animals to determine the appropriate thresholds for detecting true CaST signal (Supplementary Note 1).

Finally, although here we primarily highlight CaST as an enzymatic-based activity integrator, it is worth noting that it could also be used for Ca^2+^-dependent proximity labeling by analyzing biotinylated proteins. Thus, future studies could also enrich CaST-tagged proteins for analysis with mass spectrometry to examine activity-dependent differences in subcellular protein expression among unique neuronal cell types.

## Methods

### Cloning

All constructs used or developed in this study are listed in Supplementary Table 1. For all constructs, the vectors were double digested with restriction enzymes (New England BioLabs; NEB) following the standard digest protocols. PCR fragments were amplified using Q5 polymerase (M0494S, NEB). Both vectors and PCR fragments were purified using gel electrophoresis and gel extraction (28706, Qiagen) and were ligated using Gibson assembly (E2611S, NEB). Ligated plasmids were introduced into NEB Stable Competent *Escherichia coli* (C3040H, NEB) via a heat shock following the manufacturer’s transformation protocol. Plasmids were amplified using NEB 5-alpha Competent *E. coli* (C2987H, NEB) and Plasmid Miniprep Kit (27106, Qiagen).

### AlphaFold2 protein structure predictions

To generate AlphaFold2 (ref. ^57^) predicted structures of CaST in Fig. 1, ColabFold^56^ (v1.5.5) was used with default settings. To generate the predicted structures of the two halves of CaST in isolation, each half’s amino acid sequence was entered individually, to predict each structure separately. To generate the predicted structure of the two halves of CaST in complex, the two amino acid sequences were entered, separated by a ‘:’ to specify inter-protein chain breaks for modeling complexes (for example, heterodimers). Predicted structures were recolored using Pymol (v2.5.2).

### Mammalian cell culture and transfection

HEK293T cells (CRL-3216, American Type Culture Collection; no additional verification performed) were cultured as a monolayer in DMEM (D5796-500ML, Sigma-Aldrich), supplemented with 10% FBS (F1051-500ML, Sigma-Aldrich) and 1% (vol/vol) penicillin–streptomycin (15070063, 5,000 U ml^−1^, Life Technologies; Complete DMEM). Cells were cultured in 100-mm tissue-culture-treated dishes (353003, Falcon) and maintained in the cell culture incubator at 37 °C with humidified 5% CO_2_ and subcultured when they reached 80–90% confluence using trypsin (T2610-100ML, Sigma-Aldrich).

For immunofluorescence experiments, cells were plated in 48-well plates pretreated with 50 mg ml^−1^ human fibronectin (FC010-10MG, Millipore) and incubated for 24 h. Cells at ~80% confluency were transfected with DNA plasmids using polyethylenimine ‘Max’ according to the manufacturer’s manual (PEI Max; 24765-1, Polysciences). For CaST experiments, cells were transfected with 50 ng CD4-sTurboID (C)-M13-GFP, 20 ng CaM-V5-sTurboID (N) and 0.8 µl PEI Max per well. For CaST-IRES experiments, cells were transfected with 50 ng CD4-sTurboID (C)-M13-GFP-IRES-CaM-V5-sTurboID (N) and 0.8 µl PEI Max per well. Cells were incubated at 37 °C overnight for 15–16 h before stimulation.

For confocal experiments, cells were plated in 35-mm glass-bottom dishes (D35-14-1-N, Cellvis) pretreated with 50 mg ml^−1^ human fibronectin and incubated for 24 h. Cells at ~80% confluency were transfected with DNA plasmids using PEI Max according to the manufacturer’s manual. For CaST experiments, cells were transfected with 250 ng CD4-sTurboID (C)-M13-GFP, 125 ng CaM-V5-sTurboID (N) and 8 µl PEI Max per well. Cells were incubated at 37 °C overnight for 15–16 h before stimulation.

### HEK293T cell CaST/CaST-IRES experiments

CaST or CaST-IRES HEK cells were stimulated 15–16 h following transfection. Stimulation master mixes were made by adding CaCl_2_ (21115-100 ML, Sigma-Aldrich), ionomycin (I3909-1ML, Sigma-Aldrich) and biotin (B4639, Sigma-Aldrich; dissolved in dimethylsulfoxide as 100 mM stock) to complete DMEM. The stimulation mixtures were then added to the cell, and the cells were incubated at 37 °C for 30 min for CaST/CaST-IRES labeling. For +Ca^2+^ conditions, we used a final concentration of 5 mM Ca^2+^ and 1 µM ionomycin. For +biotin conditions, we used a final concentration of 50 µM biotin. After incubation for the indicated time, the solution was removed in each well. Cells were washed once with warm Complete DMEM and twice with warm DPBS (D8537-500ML, Sigma-Aldrich). Cells were subsequently fixed with 4% (vol/vol) paraformaldehyde (PFA; sc-281692, Santa Cruz Biotechnology) in DPBS at room temperature for 10 min followed by two washes with DPBS. After that, cells were permeabilized with ice-cold methanol at −20 °C for 8 min followed by two washes with DPBS at room temperature. Cells were incubated with blocking solution (1% wt/vol BSA; BP1600-100, Fisher Scientific, in DPBS) for 45 min at room temperature; followed by a 1-h incubation in primary antibody solution at room temperature (1:1,000 dilution mouse anti-v5; R96025, Invitrogen). Cells were washed twice with DPBS and incubated with secondary antibody (1:1,000 dilution donkey anti-mouse 568; ab175472, Abcam) and SA-647 (1:5,000 dilution; S32357, Invitrogen) for 30 min at room temperature. Cells were then washed three times with DPBS and imaged by epifluorescence microscopy (‘Immunofluorescence imaging’).

For CaST variable transfection ratio experiments, cells at ~80% confluency were transfected with different ratios of CD4-sTurboID (C)-M13-GFP and CaM-V5-sTurboID (N) using PEI Max, according to the manufacturer’s manual. Single-fragment CaST experiments were performed by transfecting cells with only one CaST fragment (either 50 ng CD4-sTb(C)-M13-GFP or 20 ng CaM-V5-sTb(N)) or both CaST fragments as positive control. Reversibility experiments were performed by treating the cells with 5 mM Ca^2+^ and 1 µM ionomycin without biotin at 37 °C for 30 min followed by two DPBS washes to wash away Ca^2+^. Then, the cells were treated with 50 µM biotin without Ca^2+^ for another 30 min. Ca^2+^ titration experiments were performed by treating the cells with different CaCl_2_ concentrations ranging from 0 to 10 mM and 1 µM ionomycin at 37 °C for 30 min. Time integration experiments were performed by treating the cells with 5 mM Ca^2+^ and 1 µM ionomycin with or without biotin at 37 °C for diverse durations ranging from 10 to 240 min. Stimulation, fixation and staining were performed as described above.

### HEK293T FLiCRE/CaST-IRES comparison experiment

Transfection of HEK293T cells with FLiCRE was performed following published protocols^6^. In detail, cells were transfected with FLiCRE constructs using PEI Max (20 ng UAS-mCherry, 30 ng CaM-TEVp, 50 ng CD4-MKII-LOV-TEVcs-Gal4 and 0.8 µl PEI Max per well in 48-well plates) at ~80% confluency and were immediately wrapped in foil and incubated overnight for 8–9 h. Transfection of CaST-IRES was performed as described above. FLiCRE-expressing cells were treated with continuous blue light ± Ca^2+^ (6 mM Ca^2+^ and 1 µM ionomycin) for 15 min at 37 °C. CaST-IRES cells were treated with biotin ± Ca^2+^ with a final concentration of 5 mM Ca^2+^, 1 µM ionomycin and 50 µM biotin and incubated at 37 °C for 15 min. After stimulation, cells were washed twice with warm DPBS and incubated with DPBS at 37 °C for 10 min. After the washes, the DPBS was removed and replaced with 200 µl Complete DMEM for further incubation. The cells were fixed immediately or 2, 4, 6 or 8 h after the stimulation. Staining was performed after all the cells were fixed following the methods described above. The biotinylated proteins (SA-647, CaST-IRES) and expression of the reporter (UAS::mCherry, FLiCRE) were imaged by fluorescence microscopy.

For extended post-transfection incubation, cells were transfected with CaST-IRES or FLiCRE constructs as described above and were incubated at 37 °C for 48 h before stimulation. Cells were stimulated, washed and incubated as described above. The cells were fixed immediately, 8 h or 24 h after the stimulation. Staining was performed after all the cells were fixed following the methods described above. The biotinylated proteins (SA-647, CaST-IRES) and expression of the reporter (UAS::mCherry, FLiCRE) were imaged by fluorescence microscopy.

### Western blot analysis of CaST

HEK293T cells expressing CaST or CaST-IRES were stimulated and labeled with biotin as described above in six-well plates and were subsequently washed three times with 1 ml DPBS. Cells were then detached from the well by gently pipetting 1 ml of ice-cold DPBS onto the cells and were pelleted by centrifugation at 300*g* at 4 °C for 3 min. The resulting supernatant was removed, and the pellet was lysed on ice for 10 min using 100 μl RIPA lysis buffer (50 mM Tris pH 8, 150 mM NaCl, 0.1% SDS, 0.5% sodium deoxycholate and 1% Triton X-100) supplemented with protease inhibitor cocktail (P8849, Sigma-Aldrich) and 1 mM phenylmethylsulfonyl fluoride (82021, G-Biosciences). The cell lysates were clarified by centrifugation at 13,000*g* at 4 °C for 10 min. The supernatants were collected and mixed with 4× protein loading buffer. The resulting samples were boiled at 95 °C for 10 min before separation on a 10% precast SDS–PAGE gel. Separated proteins on SDS–PAGE gels were transferred to a nitrocellulose membrane in transfer buffer on ice. The membrane was removed after the transfer and incubated in 5 ml Ponceau stain (0.1% wt/vol Ponceau in 5% acetic acid/water) for 5 min. The Ponceau stain was then removed with deionized water and then rinsed with TBST (Tris-buffered saline, 0.1% Tween 20). The membrane was then blocked in 5% (wt/vol) nonfat dry milk in TBST at room temperature for 1 h and washed three times with TBST for 5 min each. To detect biotinylated proteins, the membrane was incubated in 10 ml of 3% BSA/TBST (wt/vol) with 2 µl streptavidin–HRP (1:5,000 dilution; S911, Invitrogen) for 1 h at room temperature. The blot was washed three times with TBST for 5 min and was developed with Clarity Max Western ECL Substrate (Bio-Rad) for 1 min before imaging. To detect the V5 tag, the membrane was incubated in 10 ml of 3% BSA/TBST (wt/vol) with 1 µl mouse anti-V5 primary antibody (1:10,000 dilution; R96025, Invitrogen) for 1 h at room temperature. The blot was washed three times with TBST for 5 min and was incubated with 10 ml of 3% BSA/TBST (wt/vol) with 1 µl anti-mouse-HRP secondary antibody (1:10,000 dilution; 170-6516, Bio-Rad) for 30 min at room temperature. The blot was washed three times with TBST for 5 min and was developed with Clarity Max Western ECL Substrate (Bio-Rad) for 1 min before imaging. The blots were imaged on a Bio-Rad Chemi-Doc XR gel imager. The raw western blot images were quantified using Fiji/ImageJ v2.9.0 (the exact parameters used for each blot are shown in the relevant figure legends).

### AAV1/AAV2 virus production, concentration and titration

The production and concentration processes for AAV viruses were conducted following a previously reported method^5,60^. In detail, HEK293T cells at ~80% confluency in three T-150 flasks were transfected with 5.2 µg AAV vector, 4.35 µg AAV1 plasmid, 4.35 µg AAV2 plasmid, 10.4 µg DF6 AAV helper plasmid and 130 µl PEI Max solution per construct and were incubated for 48 h. After the incubation, the cell culture conditioned media (supernatant) from the T-150 flasks were collected and filtered through a filter with a 0.45-µm pore size (9914-2504, Cytiva) for cultured neuron infection. The remaining HEK293T cells in the flasks were lifted using a cell scraper and were pelleted at 800*g* for 10 min for making the purified and concentrated virus. Pelleted cells were then resuspended using 20 ml of 100 mM TBS (100 mM NaCl, 20 mM Tris, pH 8.0). The surfactant, 10% sodium deoxycholate (D5670-25G, Sigma-Aldrich), was then added to the resuspended pellet to a final concentration 0.5%, along with benzonase nuclease (E1014-5KU, Sigma-Aldrich) to a final concentration of 50 units per ml and incubated for 1 h at 37 °C. Cell debris were removed by centrifugation at 2,500*g* for 15 min and the supernatant was harvested. The clarified cell lysate was loaded to a pre-equilibrated HiTrap heparin column (GE17-0406-01, Cytiva) followed by a 10 ml 100 mM TBS wash using a peristaltic pump, and then successive washes of 1 ml of 200 mM TBS (200 mM NaCl, 20 mM Tris, pH 8.0), and 1 ml of 300 mM TBS (300 mM NaCl, 20 mM Tris, pH 8.0), using a syringe. The bound virus in the column was eluted using a sequence of 1.5 ml 400 mM TBS, 3 ml 450 mM TBS and 1.5 ml 500 mM TBS (all with 20 mM Tris, pH 8.0). Eluted viruses were then concentrated by centrifugation at 2,000*g* for 2 min using a 15-ml centrifugal unit with a 100,000-Da molecular-weight cutoff (UFC910024, Sigma-Aldrich) to a final volume of 500 µl. Further concentration of viruses was achieved using a 0.5 ml centrifugal unit (UFC510024, Sigma-Aldrich) to a final volume of 100–200 µl. Titration of the concentrated viruses was performed by AAVpro Titration Kit (for Real-Time PCR version 2; 6233, Takara Bio) following the manufacturer’s protocol. The standard curve was prepared using the positive control solution provided by Takara with serial dilution for absolute quantification. The titer of each viral sample was calculated in reference to the standard curve.

### Primary neuronal culture and infection

One day before neuron dissociation, 24-well plates and 35-mm glass-bottom dishes (D35-14-1-N, Cellvis) were coated with 0.1 mg ml^−1^ poly-d-lysine (P6407-5MG, Millipore) dissolved in 1× borate buffer (28341, Thermo Scientific) overnight at room temperature. Plates and dishes were washed four times with sterile deionized water and dried in the tissue culture hood on the day of neuron dissociation. Embryonic day 18 rat hippocampal tissue (SDEHP, Brain Bits) was dissociated with papain (PAP, Brain Bits) and DNase I (07469, StemCell Technologies) following the manufacturer’s instructions for neuron dissociation. Dissociated neurons were plated in poly-d-lysine-coated plates and dishes and cultured in complete Neurobasal Plus medium (A3582901, Life Technologies) supplemented with 0.01% (vol/vol) gentamicin (15710064, Life Technologies), 0.75% (vol/vol) GlutaMAX (35050061, Life Technologies), 2% (vol/vol) B27 Plus (A3582801, Life Technologie) and 5% (vol/vol) FBS (F1051-500ML, Sigma-Aldrich) at 37 °C, 5% CO_2_. The entire culture medium of each well and dish was removed 24 h after the plating and replaced with complete Neurobasal Plus medium supplemented with 0.01% (vol/vol) gentamicin, 0.75% (vol/vol) GlutaMAX, 2% (vol/vol) B27 Plus to stop glial growth. Subsequently, ~50% of the medium in each well was replaced every 3–4 days. At days in vitro (DIV) 6, neurons were infected with a mixture of crude supernatant of CaST-encoded AAV1/2 viruses by replacing half of the culture media. The plate and dishes were incubated for another ~2 weeks in the incubator before stimulation.

### Primary neuron CaST experiments

Rat hippocampal neurons were dissociated, plated and infected as described above. At DIV 17–22 (~2 weeks after infection), neurons were ready for stimulation. Neuron stimulation reagents including KCl (P3911-25G, Sigma-Aldrich), DOI (D101, Sigma-Aldrich) and DA (H8502, Sigma-Aldrich) were used to introduce neuron firing. A portion of the culture media from each well was taken out and saved in microcentrifuge tubes. The stimulation mixtures were generated by adding the neuron stimulation reagent and biotin solution to the saved culture media to the desired concentration. The stimulation mixtures were then added back to each well and the neurons were incubated at 37 °C for 30 min for CaST labeling. For +KCl conditions, we used a final concentration of 30 mM KCl. For +DOI conditions, we used 10 µM of DOI as the final concentration. For +DA conditions, we used a final concentration of 10 µM DA. For +biotin conditions, we used a final concentration of 50 μM biotin. Neurons were then fixed and stained as described above for HEK293T cell experiments. Time-series experiments were performed by treating the neurons with or without 30 mM KCl for 10 min or 30 min at 37 °C. Neurons were then fixed and stained as described above for HEK293T cell experiments before imaging.

### Immunofluorescence imaging

Cells and neurons were imaged immediately after fixation and staining. Fluorescence images were taken with Keyence BZ-X810 fluorescence microscope (acquisition software v1.1.2) with an 80 W metal halide lamp as the fluorescence light source and a PlanApo ×10 air objective lens (NA 0.45). Expression of CaST was visualized by GFP using a 470/40-nm excitation filter and a 525/50-nm emission filter, and Alexa Fluor 568 using a 545/25-nm excitation filter and a 605/70-nm emission filter. Biotinylated proteins were labeled by SA-647 and visualized using a 620/60-nm excitation filter and a 700/75-nm emission filter. Images were analyzed by custom scripts in Fiji/ImageJ v2.9.0 and MATLAB vR2020b (‘Analysis of CaST immunofluorescence’).

Confocal imaging was performed using a Carl Zeiss LSM 800 confocal microscope equipped with 488-, 561- and 640-nm lasers, and a ×63 oil immersion objective (NA 1.4). For high-resolution confocal imaging, 35-mm glass-bottom dishes with a 14-mm micro-well and cover glass with thickness no. 1.5 (0.16 mm–0.19 mm) were used. Fluorescence images were collected with a 512 × 512-pixel resolution and with a pixel dwell time of 1.03 μs per pixel. Images were acquired using a PMT detector and emission filter ranges of 450–575 nm, 450–640 nm and 645–700 nm for EGFP, Alexa Fluor 568 and Alexa Fluor 647 detection, respectively, for best signal. All images were collected and processed using ZEN software v2.3 (Carl Zeiss).

### Analysis of CaST immunofluorescence

For CaST fluorescence characterization, we analyzed all cells in the FOV that expressed CaST (assessed using the GFP channel). To accomplish this, the GFP images across all conditions of a given experiment (calcium-treated and non-treated) were pseudo combined into one super FOV, so that the exact same cell-detection threshold was applied equally to all images (using the ‘cell-segm’ automated thresholding script^61^). This ensured no bias in the detection of GFP^+^ cells across conditions. The cell masks were then applied to the original GFP and SA-647 images, so that the GFP and SA-647 cell fluorescence could be calculated for cells belonging to an individual FOV.

To ensure reproducibility, typically 6–12 FOVs were imaged for a given experimental condition. We reported both the raw GFP and raw SA-647 fluorescence of all cells pooled across all FOVs in a scatter plot. These raw scatter plots illustrate that the SA-647 labeling in calcium-treated cells is higher than the SA-647 labeling observed in untreated cells across generally all GFP expression levels of the tool (the entire *x* axis).

To make a quantitative comparison of this data that matched the standards previously reported for evaluating similar tools (such as FLARE^5^, FLiCRE^6^ and Cal-Light^7^), we calculated the mean SA-647/GFP cell ratios found for each FOV. This analysis can normalize for any difference in expression levels of the tool across cells and conditions. Matching the standards of these previously published works, the background SA-647 autofluorescence in the epifluorescence images was subtracted from every cell for each FOV before taking the ratio to the GFP fluorescence values (autofluorescence was calculated as the mean pixel value of the entire image, excluding pixels that corresponded to cell masks). See Supplementary Fig. 2 for an example of each step of this analysis pipeline, along with appropriate summary data.

### Neuron viability analysis

Neuron viability assays were performed using DRAQ7 dye (D15105, Invitrogen). Rat hippocampal neurons were dissociated, plated as described above and were infected with crude supernatant AAV1/2 viruses for either the complete CaST, or only one CaST fragment (CD4-sTb(C)-M13-GFP) as a DRAQ7-negative control, at DIV 6. At DIV 19, neurons were stained with DRAQ7 solution with a final concentration of 3 µM for 10 min at 37 °C, protected from light. After staining, neurons were washed with DPBS, fixed with 4% PFA, and imaged in DPBS. For the DRAQ7-positive control, neurons expressing complete CaST constructs were fixed with 4% PFA and were permeabilized with methanol for 8 min at −20 °C at DIV 19. Neurons were then incubated with DRAQ7 dye solution for 10 min at 37 °C, protected from light and were washed and imaged using fluorescence microscopy.

### Calcium imaging in cultured neurons

Rat hippocampal neurons were dissociated, plated as described above and were infected with a mixture of crude supernatant of CaST-encoded and RCaMP2-encoded AAV1/AAV2 viruses by replacing half of the culture media at DIV 6. The plate and dishes were incubated for another ~2 weeks in the incubator before stimulation. For simultaneous RCaMP2 recording and CaST labeling characterization, mild neuronal stimulation was introduced by replacing half of the volume of the neuron culture media, and biotin was also introduced at a final concentration of 50 µM for CaST labeling. Calcium activity of each RCaMP2-positive neuron was continuously recorded for 5 min after stimulation using the Keyence microscope. Neurons were treated with biotin for a total of 30 min at room temperature. After stimulation, neurons were washed, fixed and stained as described above. The same FOV recorded during RCaMP2 imaging was then reidentified, and images showing CaST expression and labeling were captured.

For calcium imaging during drug treatment, neurons were infected with RCaMP2-encoded AAV1/2 virus at DIV 6. The neurons were incubated for another ~2 weeks in the incubator before stimulation. KCl, DA and DOI stimulations were induced as described above. Calcium activity of each RCaMP2-positive neuron was continuously recorded for 1 min before and 1 min after stimulation using the Keyence microscope.

The mean RCaMP2 FOV during the recording was input to Cellpose^62^ (v2.2.3) to identify masks corresponding to individual neurons. Pixels corresponding to individual cell masks were then analyzed in Fiji to obtain the fluorescence time series of all neurons in the FOV, and values were imported into MATLAB vR2020b for further analysis. The time series of each neuron was reported either as d*F/F* (Extended Data Fig. 6), or as a *z*-score relative to the baseline recording (Extended Data Fig. 8). d*F/F* was calculated as (*F*_t _− *F*_b_)/*F*_b_, where *F*_b_ is the 2nd percentile of each cell’s fluorescence time series. *z*-scores were calculated in MATLAB using the ‘zscore’ function. To quantify the mean RCaMP2 activity following mild stimulation, the ‘findpeaks’ function was used in MATLAB, and the detected peak heights were averaged for each neuron’s activity trace (Extended Data Fig. 6). To quantify the mean peak height following the drug-induced baseline increase following calcium influx, the maximum of each cell’s calcium trace during the post-stimulation period was reported (Extended Data Fig. 8).

### Optogenetic stimulation for CaST labeling in cultured neurons

Rat hippocampal neurons were dissociated and plated as described above and were infected with a mixture of crude supernatant of CaST- and bReaChES-encoded AAV1/AAV2 viruses by replacing half of the culture media at DIV 6 and were immediately wrapped in foil. At DIV 18, dishes with CaST and bReaChES coexpressing rat hippocampal neurons were covered on the bottom with black tape to block the orange stimulation light (M595F2, Thorlabs, 5.67 mW per mm^2^). A ~1-mm slit was left open allowing spatially targeted bReaChES stimulation. Biotin was introduced at a final concentration of 50 µM for CaST labeling. Orange light was cycled every 6.5 s with 2 s on and 4.5 s off. The total stimulation was 30 min long, using 5-ms-long pulses delivered at 20 Hz during the ‘on’ cycle. Glutamate receptor antagonists APV and NBQX were added at a final concentration of 50 µM and 20 µM, respectively, at the time of light stimulation to reduce synchronized neuron firing across the entire dish. After stimulation, neurons were washed, fixed and stained as described above. Images were taken using the Keyence BZ-X810 with tile scan mode.

### Mouse animal models

All experimental and surgical protocols were approved by the University of California, Davis, Institutional Animal Care and Use Committee. For CaST experiments, 5–7-week-old male and female wild-type C57BL/6J (Jackson Laboratory Strain, 000664) mice were used. Mice were maintained on a 12-h reverse light–dark cycle (lights on at 21:00) at 22 °C and 40–60% humidity, group-housed with same-sex cage mates and given ad libitum access to food and water.

### Mouse stereotaxic surgeries

Briefly, mice were maintained under anesthesia with 1.5–2% isoflurane and placed in a stereotaxic apparatus (RWD) on a heating pad. The fur on the top of the skull was removed and antiseptic iodine and 70% alcohol were used in alternation to clean the scalp. Sterile ocular lubricant (Dechra) was administered to the eyes of the mice to protect them from drying out. A midline scalp incision was made, and 0.1% hydrogen peroxide was applied to the skull. A craniotomy was made above the injection site. Virus was then injected into the targeted region using a 33-gauge beveled needle (WPI) and a 10 μl Hamilton syringe controlled by an injection pump (WPI). For all surgeries, 1,000 nl of virus was injected into the targeted mPFC brain region (coordinates: ML, ±0.5; AP, +1.98; DV, −2.25) at a rate of 150 nl min^−^^1^.

For 2P imaging surgeries, 1,000 nl of AAV5-CaMKIIa-GCaMP6f was injected into mPFC (diluted 1:1 in DPBS; Addgene, 100834-AAV5, 2.2 × 10^12^ viral genomes per ml titer). A 1-mm diameter GRIN lens (Inscopix) was then implanted above the mPFC (ML, ± 0.5; AP, +1.98; DV, −2.05). Implants and custom stainless-steel headplates were secured to the skull using a dental adhesive and cement system (Pentron, C&B metabond). For CaST surgeries, 500 nl of a 1:1 ratio of the two homemade CaST viruses was injected into the mPFC (AAV2/1-Syn-CD4-sTb(C)-M13-GFP, 4.85 × 10^9^ copies per ml; AAV2/1-Syn-CaM-V5-sTb(N), 4.79 × 10^9^ copies per ml). Once the surgery was completed, the incision was closed with tissue adhesive (GLUture). Mice received a dose of 3.25 mg per kg body weight EthiqaXR for pain recovery and were revived in a new, clean cage placed on a heating pad.

### 2P Ca^2+^ imaging in mice

2P Ca^2+^ imaging was performed using a commercial microscope (2P+, Bruker) and a ×16, 0.8 NA objective (MRP07220, Nikon). A tunable infrared femtosecond pulse laser set to 920 nm (Coherent, Discovery TPC) was used for excitation, and fluorescence emission was collected using a GaAsP PMT (H10770PB-40, Hamamatsu). The excitation laser was directed by galvo scanners sampling 512 × 512 pixels. Each image was captured at 2 Hz. The imaging FOV was 448 × 448 μm (optical zoom of ×2.5). Data were collected using the PrairieView v5.6 software and analyzed using Suite2P^63^ (v0.14.3) and custom MATLAB vR2020b scripts. Fluorescence values corresponding to each cell mask output from Suite2P were then averaged to create a fluorescence time series for each neuron. Each neuron’s trace was *z*-scored (‘zscore’ function in MATLAB) and smoothed using a 5-s sliding window (‘smooth’ function in MATLAB). We calculated the difference between each neuron’s 10-min psilocybin activity trace and 10-min saline activity trace (‘psilocybin minus saline’ activity trace). We then took the average of this difference and ranked cells according to this ‘Avg diff’ value. We considered ‘activated’ neurons to be those with an Avg diff *z*-score value > 0.05, and ‘inhibited’ neurons to be those with an Avg diff *z*-score value < −0.05.

### CaST experiments in mice

Mice were handled and given USP-grade saline (0.9%) injections for three consecutive days before biotin labeling. After a week of viral expression, experimental mice were given two injections, one 24 mg per kg body weight i.p. injection of diluted biotin solution (B4639, Sigma-Aldrich; dissolved in dimethylsulfoxide as a 10 mM stock solution) and one 3 mg per kg body weight i.p. injection of psilocybin (synthesized as previously described^36^) dissolved in saline. Control mice were injected with biotin solution and saline (5 ml per kg body weight, i.p.). Mice were placed in separated clean cages following the injection and were euthanized 1 h after i.p. injections. A subset of mice was video recorded for head-twitch data acquisition following injections. The number of HTRs during the first 20 min of video recording was quantified, manually scored by a blinded experimenter.

After biotin labeling, mice were perfused with ice-cold PBS, followed by 4% PFA, and brains were collected and stored in 4% PFA overnight at 4 °C. The next day, brains were switched out from PFA and stored in PBS until slicing. Next, 60-μm slices were collected from the mPFC and placed in wells with PBS and stored in 4 °C. Slices were washed with PBS-T for 2 min (3×), then blocked in 5% normal donkey serum (017-000-121, Jackson ImmunoResearch) and 0.3% Triton X-100 (T9284-100ML, Sigma-Aldrich; in PBS-T) for 1 h at room temperature. Slices were stained with SA-647 (1:1,000 dilution; S32357, Invitrogen) in 5% NDS/PBS-T for 1.5 h at room temperature. Slices were washed with PBS-T for 5 min at room temperature and then mounted with DAPI-Fluoromount-G (0100-20, SouthernBiotech) to adhere coverslips. Images at a magnification of ×40 were taken on the BZ-X810 Keyence fluorescence microscope and were analyzed as described above for ‘Analysis of CaST immunofluorescence’*.*

### c-Fos staining of mouse brain slices

Mice were injected with CaST and treated with saline or psilocybin as described above. Following perfusion of the brain and slicing, a subset of slices was saved for c-Fos-only staining. Slices were washed in PBS-T (1% Triton X-100 in PBS) for 2 h and placed on shaker at room temperature. Slices were blocked in 1% BSA/PBS-T for 1 h at room temperature, and then stained with 300 µl of rat anti-c-Fos primary antibody (226017, Synaptic Systems) at a 1:1,000 dilution in 1% BSA/PBS-T overnight on a shaker at room temperature. The next day, slices were washed with PBS-T for 20 min, three times, and placed on a shaker at room temperature. Slices were then stained with 300 µl of anti-rat-Alexa Fluor 568 secondary antibody (A78946, Invitrogen) at a 1:500 dilution in 1% BSA/PBS-T for 2 h on a shaker at room temperature. Slices were washed with PBS-T for 20 min, two times, and placed on a shaker at room temperature. Slices were mounted in DAPI-Fluoromount-G to adhere coverslips. For simultaneous CaST staining, the same procedure was followed, except SA-647 was also included during the secondary antibody incubation (1:1,000 dilution; S32357, Invitrogen). Images at a magnification of ×40 were taken on the BZ-X810 Keyence fluorescence microscope, and were analyzed using an automated cell-detection method in MATLAB (cell-segm)^61^ for each channel individually.

### Statistical analyses

Statistical analyses including Mann–Whitney *U* test, ordinary one-way ANOVA, two-way ANOVA, Pearson correlation analysis and Wilson/Brown ROC curve analysis were performed in GraphPad Prism v9.0 (GraphPad Software). The D’Agostino–Pearson test for normality was performed in Prism before using any parametric statistical tests. Significance was defined as a *P < 0.05, **P < 0.01 and ***P < 0.001 for the defined statistical test (NS, *P* ≥ 0.05). All experiments performed in this study were independently replicated at least twice.

### Reporting summary

Further information on research design is available in the Nature Portfolio Reporting Summary linked to this article.

## Online content

Any methods, additional references, Nature Portfolio reporting summaries, source data, extended data, supplementary information, acknowledgements, peer review information; details of author contributions and competing interests; and statements of data and code availability are available at 10.1038/s41592-024-02375-7.

## Supplementary information

Supplementary Information Supplementary Figs. 1–3, Table 1 and Note 1.

Reporting Summary

Supplementary Data 1 Source data for Supplementary Fig. 1.

Supplementary Data 2 Source data for Supplementary Fig. 2.

Supplementary Data 3 Source data for Supplementary Fig. 3.

## Source data

Source Data Fig. 1 Statistical source data.

Source Data Fig. 2 Statistical source data.

Source Data Fig. 3 Statistical source data.

Source Data Fig. 4 Statistical source data.

Source Data Fig. 5 Statistical source data.

Source Data Fig. 6 Statistical source data.

Source Data Extended Data Fig. 1 Statistical source data.

Source Data Extended Data Fig. 2 Statistical source data and unprocessed western blots.

Source Data Extended Data Fig. 3 Statistical source data.

Source Data Extended Data Fig. 4 Statistical source data.

Source Data Extended Data Fig. 5 Statistical source data.

Source Data Extended Data Fig. 6 Statistical source data.

Source Data Extended Data Fig. 7 Statistical source data.

Source Data Extended Data Fig. 8 Statistical source data.

Source Data Extended Data Fig. 9 Statistical source data.

Source Data Extended Data Fig. 10 Statistical source data.

## Data Availability

Plasmids and associated DNA sequences generated in this study are available on Addgene (catalog nos. 219779–219784; https://www.addgene.org/christina_kim/). There are no restrictions on data availability. Source data are provided with this paper.

## Code availability

Custom MATLAB scripts used to analyze images are freely available under The MIT license at https://github.com/tinakimlab/CaST-analysis-scripts/.
