# Supplementary material for: Rapid, biochemical tagging of cellular activity history in vivo
Source: Nat Methods. 2024 Aug 5;21(9):1725–35. doi: 10.1038/s41592-024-02375-7 (PMC11399108; doi:10.1038/s41592-024-02375-7)
Supplement: Supplementary file 2 — Reporting Summary [file 41592_2024_2375_MOESM2_ESM.pdf]

nature portfolio

Corresponding author(s): Christina Kim

Last updated by author(s): Apr 23, 2024

Reporting Summary

Nature Portfolio wishes to improve the reproducibility of the work that we publish. This form provides structure for consistency and transparency in reporting. For further information on Nature Portfolio policies, see our Editorial Policies and the Editorial Policy Checklist.

Statistics

For all statistical analyses, confirm that the following items are present in the figure legend, table legend, main text, or Methods section.

n/a	Confirmed	
☐	☒	The exact sample size (*n*) for each experimental group/condition, given as a discrete number and unit of measurement
☐	☒	A statement on whether measurements were taken from distinct samples or whether the same sample was measured repeatedly
☐	☒	The statistical test(s) used AND whether they are one- or two-sided<br>*Only common tests should be described solely by name; describe more complex techniques in the Methods section.*
☐	☒	A description of all covariates tested
☐	☒	A description of any assumptions or corrections, such as tests of normality and adjustment for multiple comparisons
☐	☒	A full description of the statistical parameters including central tendency (e.g. means) or other basic estimates (e.g. regression coefficient) AND variation (e.g. standard deviation) or associated estimates of uncertainty (e.g. confidence intervals)
☐	☒	For null hypothesis testing, the test statistic (e.g. *F*, *t*, *r*) with confidence intervals, effect sizes, degrees of freedom and *P* value noted<br>*Give P values as exact values whenever suitable.*
☒	☐	For Bayesian analysis, information on the choice of priors and Markov chain Monte Carlo settings
☒	☐	For hierarchical and complex designs, identification of the appropriate level for tests and full reporting of outcomes
☐	☒	Estimates of effect sizes (e.g. Cohen's *d*, Pearson's *r*), indicating how they were calculated

Our web collection on statistics for biologists contains articles on many of the points above.

Software and code

Policy information about availability of computer code

Data collection	Data were collected using commercial software associated with imaging hardware (BZ-X810 image acquisition software v1.1.2 from Keyence; Zen software v2.3 from Carl Zeiss; Prairie View software v5.6 from Bruker).
Data analysis	Data were analyzed using custom scripts in MATLAB vR2020b, Fiji/ImageJ v2.9.0, Suite2p v0.14.3, Cellpose v2.2.3, ColabFold v1.5.5, and Pymol v2.5.2. Statistical analysis was performed using GraphPad Prism v9.0. Custom MATLAB scripts used to analyze images are available at: https://github.com/tinakimlab/CaST-analysis-scripts.

For manuscripts utilizing custom algorithms or software that are central to the research but not yet described in published literature, software must be made available to editors and reviewers. We strongly encourage code deposition in a community repository (e.g. GitHub). See the Nature Portfolio guidelines for submitting code & software for further information.

Data

Policy information about availability of data

All manuscripts must include a data availability statement. This statement should provide the following information, where applicable:

- Accession codes, unique identifiers, or web links for publicly available datasets
- A description of any restrictions on data availability
- For clinical datasets or third party data, please ensure that the statement adheres to our policy

Plasmids and associated DNA sequences generated in this study are available on Addgene (catalog nos. 219779–219784; https://www.addgene.org/Christina_Kim/). There are no restrictions on data availability. Source data are provided with this paper.

Research involving human participants, their data, or biological material

Policy information about studies with human participants or human data. See also policy information about sex, gender (identity/presentation), and sexual orientation and race, ethnicity and racism.

Reporting on sex and gender	N/A
Reporting on race, ethnicity, or other socially relevant groupings	N/A
Population characteristics	N/A
Recruitment	N/A
Ethics oversight	N/A

Note that full information on the approval of the study protocol must also be provided in the manuscript.

Field-specific reporting

Please select the one below that is the best fit for your research. If you are not sure, read the appropriate sections before making your selection.

☒ Life sciences ☐ Behavioural & social sciences ☐ Ecological, evolutionary & environmental sciences

For a reference copy of the document with all sections, see nature.com/documents/nr-reporting-summary-flat.pdf

Life sciences study design

All studies must disclose on these points even when the disclosure is negative.

Sample size	No calculations were performed to pre-determine sample size. Instead, sample sizes were chosen based on prior studies utilizing similar methods to characterize activity-dependent sensors (e.g., pubmed ref PMID 33308478, and 28650461).
Data exclusions	No data were excluded from the study.
Replication	All data were replicated at least twice in this study, and often by independent investigators.
Randomization	Identical well plates were randomly assigned to each experimental condition. Identical cage-mate mice were randomly assigned to each experimental condition.
Blinding	All image acquisition and image analysis was performed using fully automated means; as such, the experimenter had no control over the data collection/which FOVs were imaged or analyzed. Mouse histology imaging was performed by a separate experimenter blinded to the experimental conditions.

Reporting for specific materials, systems and methods

We require information from authors about some types of materials, experimental systems and methods used in many studies. Here, indicate whether each material, system or method listed is relevant to your study. If you are not sure if a list item applies to your research, read the appropriate section before selecting a response.

Materials & experimental systems

n/a	Involved in the study
☐	☒ Antibodies
☐	☒ Eukaryotic cell lines
☒	☐ Palaeontology and archaeology
☐	☒ Animals and other organisms
☒	☐ Clinical data
☒	☐ Dual use research of concern
☒	☐ Plants

Methods

n/a	Involved in the study
☒	☐ ChIP-seq
☒	☐ Flow cytometry
☒	☐ MRI-based neuroimaging

Antibodies

Antibodies used

Mouse anti-v5 (Invitrogen, R96025), donkey anti-mouse 568 (Abcam, ab175472), streptavidin-Alexa Fluor647 (Invitrogen, S32357), anti-mouse-HRP (Bio-Rad, 170-6516), streptavidin-HRP (Invitrogen, s911), Rat anti-cFos (Synaptic systems, 226017)

Validation

Mouse anti-v5: validated at ThermoFisher for IHC (72 publications) and WB (631 publications); Donkey anti-mouse 568: validated at ThermoFisher in 9 published figures; streptavidin-Alexa Fluor647: validated at ThermoFisher (18 publications); anti-mouse-HRP: validated at BioRad using KO/siRNA/IP-MS validation; streptavidin-HRP: validated at ThermoFisher (69 publications); Rat anti-cFos: validated at Synaptic Systems in 11 published studies.

Eukaryotic cell lines

Policy information about cell lines and Sex and Gender in Research

Cell line source(s)

HEK293T, line CRL-3216 from ATCC.

Authentication

No additional cell line authentication was performed.

Mycoplasma contamination

This cell line was not tested for Mycoplasma contamination.

Commonly misidentified lines
(See ICLAC register)

No commonly misidentified cell lines were used in this study.

Animals and other research organisms

Policy information about studies involving animals; ARRIVE guidelines recommended for reporting animal research, and Sex and Gender in Research

Laboratory animals

All experimental and surgical protocols were approved by the University of California, Davis, Institutional Animal Care and Use Committee. For CaST experiments, 5-7 week old male and female wild-type C57BL/6J (Jackson Laboratory Strain 000664) mice were used. Mice were maintained on a 12h reverse light-dark cycle (lights on at 9PM) at 22°C and 40-60% humidity, group-housed with same-sex cage mates and given ad-lithium access to food and water.

Wild animals

No wild animals were used in this study.

Reporting on sex

Both male and female mice were used, and randomly interspersed across groups. We did not consider sex as a biological variable in this study and did not separate data based on sex.

Field-collected samples

No field-collected samples were used in this study.

Ethics oversight

All experimental and surgical protocols were approved by the University of Caliornia, Davis, Institutional Animal Care and Use Committee.

Note that full information on the approval of the study protocol must also be provided in the manuscript.
