# Supplementary figures and images for: Rapid, biochemical tagging of cellular activity history in vivo
Source: Nat Methods. 2024 Aug 5;21(9):1725–35. doi: 10.1038/s41592-024-02375-7 (PMC11399108; doi:10.1038/s41592-024-02375-7)

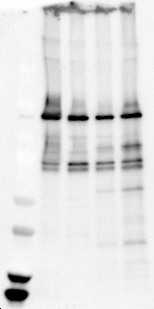

Supplement: Supplementary file 13 — Statistical source data and unprocessed western blots. [file 41592_2024_2375_MOESM13_ESM.zip › Zhang_Unmodified_Gels_ED_Fig2A.tif]

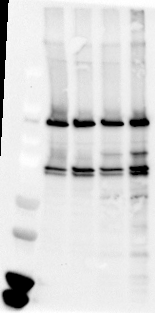

Supplement: Supplementary file 13 — Statistical source data and unprocessed western blots. [file 41592_2024_2375_MOESM13_ESM.zip › Zhang_Unmodified_Gels_ED_Fig2B.tif]

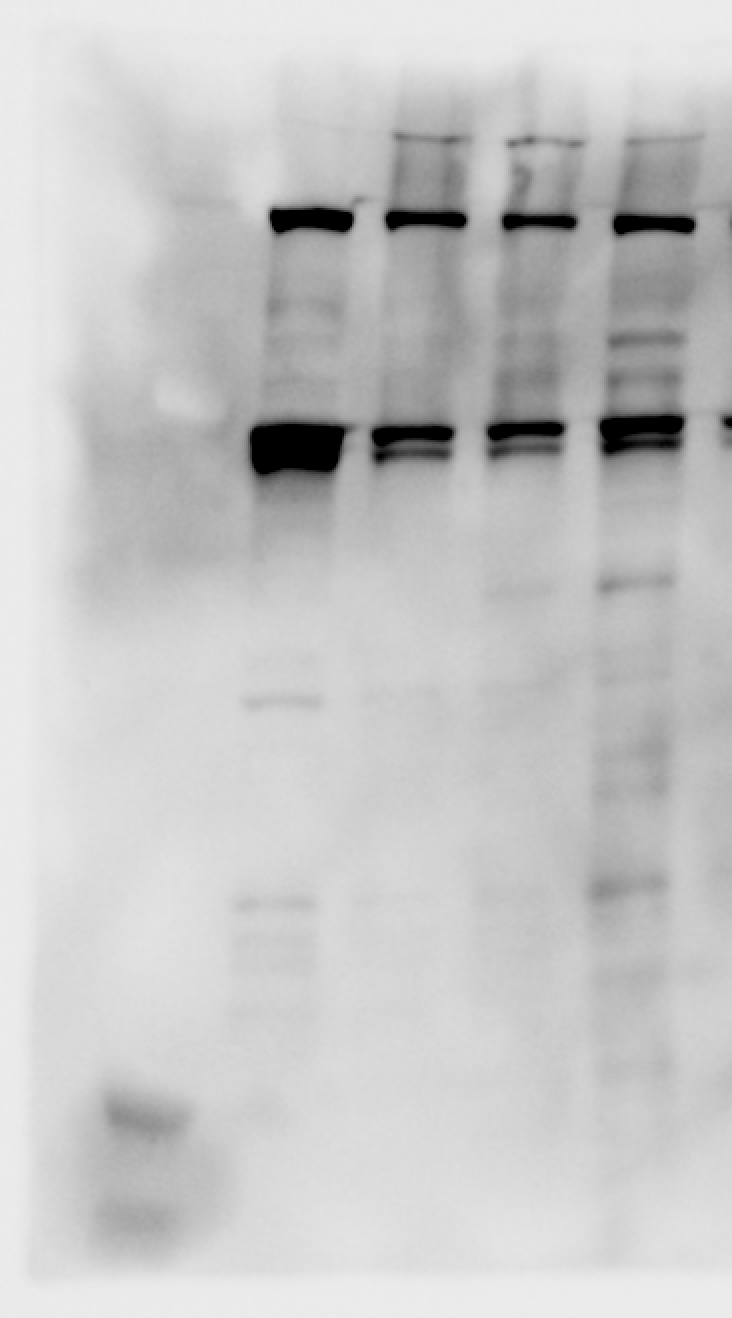

Supplement: Supplementary file 13 — Statistical source data and unprocessed western blots. [file 41592_2024_2375_MOESM13_ESM.zip › Zhang_Unmodified_Gels_ED_Fig2E.tif]
